# How to improve the potential of microalgal biostimulants for abiotic stress mitigation in plants?

**DOI:** 10.3389/fpls.2025.1568423

**Published:** 2025-04-22

**Authors:** Bram Vangenechten, Barbara De Coninck, Johan Ceusters

**Affiliations:** ^1^ Research Group for Sustainable Crop Production & Protection, Division of Crop Biotechnics, Department of Biosystems, KU Leuven, Geel, Belgium; ^2^ KU Leuven Plant Institute (LPI), KU Leuven, Leuven, Belgium; ^3^ Plant Health and Protection Laboratory, Division of Crop Biotechnics, Department of Biosystems, KU Leuven, Leuven, Belgium; ^4^ Centre for Environmental Sciences, Environmental Biology, UHasselt, Diepenbeek, Belgium

**Keywords:** biostimulant, abiotic stress, microalgae, stress tolerance, sustainable agriculture, drought, salt, heat

## Abstract

Abiotic stress is among the most critical factors limiting crop productivity worldwide and its importance is further exacerbated by climate change. In recent years, microalgal biostimulants have gained attention for their potential to enhance plant resilience towards abiotic stress. However, significant hurdles still persist, particularly regarding the unknown modes of action of microalgal biostimulants, which is a concern for stringent regulatory requirements and product reliability. The aim of this review is to improve the potential of microalgal biostimulants for abiotic stress mitigation in plants by addressing different key parameters shaping the efficacy of microalgal biostimulants, encompassing cultivation approaches, extraction techniques, and application methods. Furthermore, it also highlights how microalgal biostimulants modulate plant morphology, physiology and biochemistry under drought, salinity, and heat stress—three predominant stressors anticipated to intensify under climate change. Notably, these biostimulants consistently enhance drought stress tolerance by improving biomass accumulation, nutrient uptake, and water use efficiency through enhanced photosynthesis and stomatal regulation. These effects are largely driven by the accumulation of osmoprotectants and antioxidant compounds. In contrast, salt stress mitigation is highly species-dependent, with some microalgae enhancing stress tolerance through osmoprotectant and antioxidant accumulation, while others reduce these compounds, potentially lowering stress perception via unknown mechanisms. Despite the significance of the abiotic stress, heat stress mitigation by microalgal biostimulants remains an underexplored research area. Additionally, indirect applications of microalgae—ranging from biotechnological innovations to desalination—underscore the broader potential of these organisms in agricultural resilience. Collectively, this review identifies three key gaps in the existing literature—the diversity gap, the practical gap, and the research gap—while outlining promising avenues for future research in microalgal biostimulant development.

## 1 Introduction

The Earth’s human population is estimated to rise to 9.7 billion by 2050 (FAO et al., 2023). To meet the needs of this large population, food production needs to increase by 35% to 56% (van Dijk et al., 2021). This challenge is further compounded by current agricultural practices, which contribute to environmental degradation and climate change (Reganold and Wachter, 2016; Crist et al., 2017; Arora, 2019). Agriculture, which occupies about 44% of the world’s habitable land, is directly affecting ecosystem functioning and biodiversity due to ongoing expansion and intensification (Ritchie and Roser, 2019; Barros-Rodríguez et al., 2021; Beillouin et al., 2021; Lal, 2021). Additionally, the agricultural sector significantly contributes to greenhouse gas emissions, exacerbating climate change and, in turn, affecting the sector itself (Mbow et al., 2019). Climate change intensifies the effects of abiotic stress on crops through the occurrence of more frequent and severe weather events like heatwaves, droughts, and floods (Rong et al., 2021; Clarke et al., 2022). Under intense warming scenarios, the yields of several major crops are projected to decline by 11–25% by the end of the century (Wing et al., 2021). Furthermore, approximately 40% of the world’s arable land is at risk of aridity, and around 20% is affected by soil erosion due to climate change and, by extension, current agricultural practices (Prăvălie et al., 2021). Considering these issues, it is evident that abiotic stresses, particularly drought, salinity, and extreme temperatures, are an intensifying problem that will exacerbate over time (Zhang et al., 2023). There is an urgent need for a new green revolution that prioritizes sustainability and abiotic stress tolerance in crop production methods.

Tackling these challenges necessitates a global paradigm shift in traditional agriculture practices. Research efforts worldwide are increasingly focused on developing innovative solutions to enhance abiotic stress tolerance and sustainability in crop production. Prominent approaches include the use of precision digital tools (Kumar et al., 2024), interbreeding with crop wild relatives (Kapazoglou et al., 2023), and advanced genome-editing techniques (Zafar et al., 2020). Another promising solution lies in the application of renewable bio-active resources, specifically biostimulants (Mutale-joan et al., 2020; Parmar et al., 2023; Prisa and Spagnuolo, 2023). Biostimulants are substances or microorganisms derived from a wide variety of sources, including inorganic compounds, living microorganisms, algae, and plant extracts. They are characterized by their ability to stimulate natural processes in plants independently of their intrinsic nutritional value. Biostimulants enhance nutrient uptake and use efficiency, improve crop quality, and increase plant resilience to abiotic stresses (du Jardin, 2015).

Microalgae have shown promising biostimulant activity and have gained increased attention in recent years. Algae are classified based on size with microalgae ranging between 0.8 µm to 0.5 mm in length and macroalgae from 0.5 mm onwards (Bello et al., 2021). Typically unicellular phototrophic organisms, microalgae can be grown in both freshwater and marine environments (Safi et al., 2014; Borowitzka, 2018; Chew et al., 2018). Because microalgae are phototrophic and have proven to be able to grow on wastewater, they represent a renewable source of biostimulants without competing with food crops for land. Moreover, they contribute significantly to CO_2_ sequestration, aiding climate change mitigation (Safi et al., 2014; Sánchez-Quintero et al., 2023; Su et al., 2023). Microalgae also possess a wide array of high-value and bioactive components, such as amino acids, phytohormones, proteins, antioxidant molecules, etc (Kapoore et al., 2021). These high-value components, whether in the form of extracts, complete biomass, or live cells of microalgae, have already been proven to exhibit significant biostimulant activity. The observed responses include enhanced plant growth, improved soil health, and increased tolerance to diverse abiotic stresses. The chemical diversity of microalgal species, shared molecular pathways with higher plants, and the ability to tailor microalgal growth conditions for specific chemical compositions underscore the high potential of microalgae in abiotic stress remediation (Colla and Rouphael, 2020; Bello et al., 2021). However, the biostimulant effects have often been shown to be quite variable (Mutale-Joan et al., 2023). Factors, such as extraction techniques, dosage, application methods and timing of application—including the time of day, the developmental stage, or the timing compared to the onset of abiotic stress (preventive, curative, or recovery)—significantly influence their effectiveness in mitigating abiotic stress (Carillo et al., 2020).

Despite various studies showcasing the potential of microalgal biostimulants in helping plants cope with drought, salinity, high temperatures, and other abiotic stresses, much of the focus has been on optimal plant growth conditions (Carillo et al., 2020; Parmar et al., 2023). The mode of action of microalgal biostimulants in abiotic stress remediation remains poorly understood, which hampers their broader acceptance and integration into modern agricultural practices and regulations. Moreover, studies often focus on specific crops at particular developmental stages and stress conditions, lacking a comprehensive overview or directive for further testing strategies.

This review aims to provide insights into the factors that influence the effectiveness of microalgal biostimulants in mitigating abiotic stresses, focusing on microalgal cultivation strategies, extraction techniques, and application methods. Building on these foundational considerations, it will present a comprehensive overview of the effects of microalgae on the three most prominent abiotic stresses, particularly drought, salinity, and heat, emphasizing the known modes of action. This review will consider all true microalgae as well as the cyanobacterium *Arthrospira platensis*. Although not a true microalga, *A. platensis* is often grouped with microalgae in the literature due to its economic importance and many shared characteristics. The primary aim of this review is to elucidate the potential of microalgal biostimulants in abiotic stress remediation and to identify gaps in the existing literature, thereby guiding future research endeavors.

## 2 Determining factors shaping the efficacy of microalgal biostimulants

The role of microalgae as biostimulants is increasingly recognized due to their diverse array of bioactive compounds, including phenolics, phytohormones, polysaccharides, and proteins (Kapoore et al., 2021). The relative concentrations of these compounds are influenced by cultivation strategies, extraction techniques, application methods, and timing. These factors play a key role in shaping the efficacy of microalgal biostimulants, offering both opportunities for optimization but also challenges for ensuring consistency. On one hand, tailoring these parameters can lead to optimization of specific compounds, while on the other, their variability can complicate comparisons of results across studies. Fundamental differences in these factors can significantly impact biostimulant activity. Therefore, highlighting that these variables influence the efficacy of microalgal biostimulants is crucial, particularly when investigating their modes of action in enhancing abiotic stress resilience.

### 2.1 Cultivation strategies

The growth and biochemical composition of microalgae during cultivation are influenced by a range of factors, including the chemical composition of the growth medium, pH, temperature, salinity, and light exposure conditions. By modulating these specific environmental parameters, it is possible to cultivate microalgae with a tailored composition, enriched in certain bioactive molecules that are of particular interest for biostimulant activity (Vuppaladadiyam et al., 2018). A prominent example is the carotenoid content of *Dunaliella salina*, a photosynthetic pigment with antioxidant properties, is significantly enhanced under high salinity conditions, reaching up to 4.5 M NaCl (Farhat et al., 2011). This salinity level is substantially higher than those used for other microalgae species such as *Chlorella vulgaris* and *Nannochloropsis salina*, which are typically cultured at lower NaCl concentrations, ranging from less than 0.5 M to approximately 1 M (Bartley et al., 2013; Keo and Kaosol, 2020). Similarly, in *Scenedesmus almeriensis*, higher CO_2_ concentrations (3% v/v) in the growth medium nearly doubled biomass production and increased lutein content, a carotenoid with potent antioxidant properties, by more than 50% compared to lower CO_2_ concentrations (0.5% v/v) (Molino et al., 2020). Light intensity is another crucial factor influencing the chemical composition of microalgae. For example, moderate light levels (400 µmol photons/m²·s) promoted carotenoid accumulation in species such as *Arthrospira maxima*, *Chlorella minutissima*, *Rhodomonas salina*, and *Nannochloropsis oceanica*. In contrast, a higher light intensity (800 µmol photons/m²·s) favored the accumulation of lipids and α-tocopherol, a fat-soluble antioxidant (Ljubic et al., 2021). Modifying these environmental parameters can alter the chemical composition of microalgae, as such influencing their biostimulant activity. For example, Ranglová et al. (2021) observed that *C. vulgaris* cultivated in a standard growth medium stimulated watercress (*Lepidium sativum*) germination. However, when cultivated in household wastewater with a markedly different chemical composition, the biostimulant activity was lost. In contrast, *Chlorella fusca* LEB 111 and *Arthrospira* sp. LEB 18 cultivated in various dilutions of dairy effluent wastewater (combined with standard cultivation medium) showed diverse effects on tomato (*Lycopersicon esculentum*) seeds and plants, depending on the nutrient composition of the medium. Microalgae cultivated in higher fractions of dairy effluent promoted germination and enhanced vegetative growth, including longer roots, greater dry weight, increased plant height, and more leaves (Gonzales Cruz et al., 2023). These findings highlight the critical role of cultivation strategies in determining the biostimulatory effects of microalgae on crop resilience and growth.

### 2.2 Extraction techniques

In addition to cultivation strategies, the extraction technique and solvents used are critical tools for enhancing the concentration of specific bioactive components in microalgal biostimulants. Various extraction techniques have been developed, each with distinct advantages and limitations. (I) Traditional mechanical extraction methods, such as bead milling, sonication, and homogenization, are widely used to disrupt cell walls and facilitate the release of intracellular compounds. The efficiency of these methods is mainly influenced by specific characteristics of the microalgal cell walls, which vary by strain, thereby impacting the yield of bioactive compounds in the final extract (Sánchez-Quintero et al., 2023). Stirk et al. (2020) explored the impact of freeze-drying combined with sonication or bead-milling on the extraction of antioxidant and biostimulant compounds from *C. vulgaris* and *Scenedesmus acutus*. Their results demonstrated that the effectiveness of these treatments varied for different microalgal species, with bead-milling enhancing the antioxidant activity in *C. vulgaris* while reducing it in *S. acutus*. The authors proposed that this variation could be attributed to the dilution of active antioxidant compounds by non-active substances or the release of inhibitory compounds during the extraction process. Furthermore, the study included a rooting bioassay on mung bean seedlings to evaluate biostimulant activity. Cut seedlings were exposed to sonicated or bead-milled microalgal extracts and subsequently transferred to water to root. The bioassay revealed that cell wall disruption techniques for these microalgae generally led to a reduced number of roots in mung bean, indicating that certain extraction methods may negatively impact biostimulant efficacy. (II) Chemical extraction methods, including acid and alkaline hydrolysis, utilize reagents such as sodium hydroxide and sulfuric acid to degrade cell walls and release bioactive compounds (Michalak and Chojnacka, 2014; Bello et al., 2021). For instance, Chovanček et al. (2023) applied two distinct extraction techniques, i.e. thermal hydrolysis with a weak solution of sulfuric acid accompanied by ultrasonication or bead-milling an aqueous extraction followed by centrifugation, to six microalgal species and tested them for biostimulant activity on *A. thaliana* and lettuce (*Lactuca sativa* L. cv. Finstar). The hydrolyzed extracts showed promising biostimulant activity, stimulating root elongation in *Arabidopsis thaliana* and increasing lettuce yield by 12-15%, in contrast to the aqueous extracts, which did not significantly enhance plant growth. Solvent-based extraction techniques, such as those using the Soxhlet apparatus, remain widely employed for extracting bioactive compounds from solid samples due to their operational simplicity and scalability (Ramluckan et al., 2014). However, these methods often require large volumes of solvents and extended processing times, which can reduce the yield of bioactive compounds. Furthermore, the application of microalgae in circular and sustainable contexts is often challenging to reconcile with the environmental impact and resource demands of solvent-based extraction techniques. (III) In contrast, novel extraction techniques, such as supercritical fluid extraction, microwave-assisted extraction, enzyme-assisted extraction, and pressurized liquid extraction, offer more efficient, cost-effective, and environmentally sustainable alternatives (Ibañez et al., 2012; Bello et al., 2021). For instance, supercritical fluid extraction of the acidophilic microalga *Coccomyxa onubensis* under optimized conditions (70°C, 40 MPa, and 50% v/v ethanol) produced an extract rich in total phenols and with high antioxidant activity (Ruiz-Domínguez et al., 2022). Similarly, Navarro-López et al. (2023) demonstrated that high-pressure homogenization led to a higher degree of hydrolysis of *Scenedesmus* sp. proteins. While these extracts show potential to enhance biostimulant activity, further investigation is required to confirm and understand their effects.

### 2.3 Application methods

Microalgal biostimulants are primarily applied to plants through three main methods: seed, foliar, or soil applications. Seed applications, such as coating and priming (controlled seed hydration through soaking or dipping), are used to enhance germination and early development, as well as to support the actions of beneficial microflora (Sharma et al., 2014; Rocha et al., 2019; Devika et al., 2021). Foliar applications, which involve the direct application of bioactive compounds to plant leaves, are hypothesized to work through entry via stomata, pores, or cuticle cracks, although the exact mechanisms are not yet fully understood (Ishfaq et al., 2022). Soil applications target the root zone, with microalgae being delivered either as a powder or in solution via irrigation. Additionally, hydroponic or aeroponic systems can incorporate microalgae as part of the nutrient solution, effectively serving as a soil application (Alvarez et al., 2021). The choice of application method is critical and depends on the specific needs of the plant, such as nutritional enhancement, yield improvement, or stress mitigation, as well as the cultivation method (e.g., direct seeding, field transplantation, or nursery growth). For instance, Puglisi et al. (2022) found that while both foliar and soil applications of a methanolic extract of *C. vulgaris* resulted in increased lettuce growth, the biochemical responses differed from each other. Soil application primarily impacted carbon metabolism by significantly enhancing the activity of enzymes involved in the Krebs cycle, such as citrate synthase and malate dehydrogenase. In contrast, foliar treatment influenced nitrogen metabolism by stimulating the activity of enzymes like glutamine synthetase and glutamate synthase. Although different studies provide specific insights, there is no universal guideline for the optimal application method of microalgae on different crops (Renuka et al., 2018; Parmar et al., 2023; Sánchez-Quintero et al., 2023). Some studies have already explored the optimal application method for commonly used microalgal biostimulants on specific crops. For example, comparisons of soil and foliar applications of *C. vulgaris* on tomato have shown that soil application is more effective in enhancing growth, yield, and fruit quality than foliar application (Özdemir et al., 2016; Suchithra et al., 2022). However, the number of studies examining this remains limited. Another significant issue pertains to the timing, frequency, and concentration of applications, for which no clear consensus has been reached in literature (Sánchez-Quintero et al., 2023). Foliar application remains the most commonly used method for applying microalgae across various plant species, including food crops, flowers, and trees (Oancea et al., 2013; Plaza et al., 2018; Kanchan et al., 2019; Bello et al., 2021). When applying a foliar treatment, it is important to consider the time of application, as biostimulant uptake is presumably higher in the morning when stomata are open, and relative humidity is high (Berry et al., 2019).

## 3 Thirst for survival: microalgal interventions for drought-stressed crops

### 3.1 Introduction

Drought stress, or water deficit, is a major threat for global crop production and food security. It is not just a problem in arid regions, but due to climate change and poor water-use policies, it is a phenomenon that is present all over the world (Pokhrel et al., 2021). Drought stress affects the plant’s ability to absorb water and nutrients, leading to significant reductions in biomass and yield. Wang et al. (2020) reported that severe drought years over the past five decades in Northeast China reduced maize yields by 14.0% and soybean yields by 21.8%. Similarly, Pinke et al. (2024) found that drought-related yield losses across croplands in the European Union amounted to 25–30 billion euros in 2022, an exceptionally dry year for Europe. On a global scale, Kim et al. (2019) found that 75% of the global harvested areas for four major crops (maize, rice, soy, and wheat) with a total of 454 million hectares, experienced drought-induced yield losses between 1983 and 2009. These losses led to a cumulative global production deficit valued at 166 billion U.S. dollars. It is obvious that there is a significant environmental and economic incentive to reduce the adverse effects of drought stress on crop production, for example with microalgal biostimulants.

**Figure 1:**
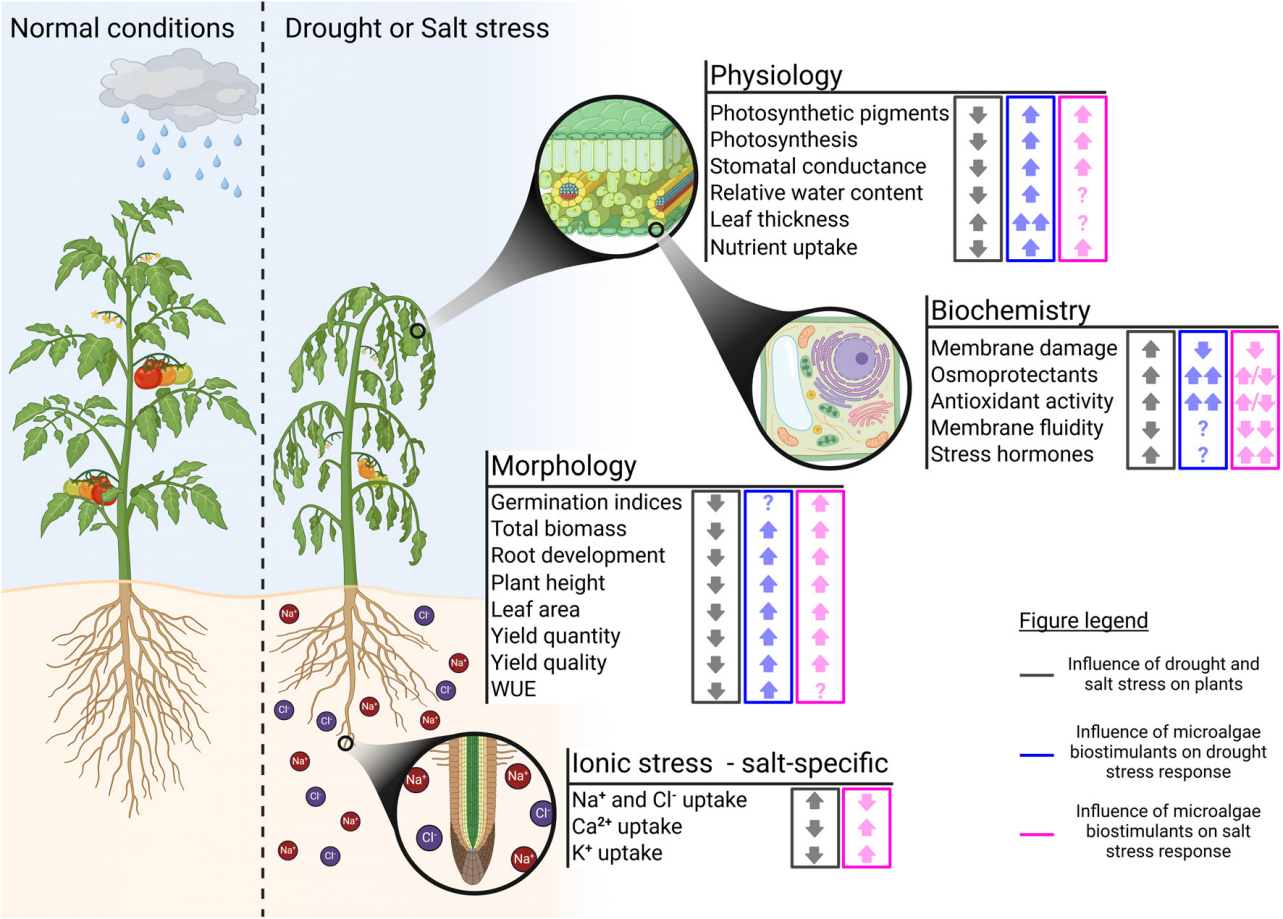
Visual overview of the morphological, physiological, and biochemical effects of microalgal biostimulants on plants exposed to drought or salt stress. Gray: influence of drought and salt stress on the plant parameters listed. Blue: influence of microalgal biostimulants on drought stress response. Pink: influence of microalgal biostimulants on salt stress response. ↑, increase; ↑↑, strong increase; ↓, decrease; ↓↓, strong decrease; ↑/↓, contradiction in literature; ?, unknown; WUE, water use efficiency (figure created with BioRender).

Drought stress affects plants at multiple levels, from visible morphological changes to underlying physiological and biochemical processes. While morphological symptoms such as reduced growth and yield are immediately evident, drought also induces critical but less visible changes in photosynthesis, osmoregulation, and hormonal balance (Yang et al., 2021). Diverse microalgal treatments have shown the ability to mitigate these effects across different levels. The following sections will explore the specific impacts of drought on plant morphology, physiology, and biochemistry, and discuss how microalgal applications can enhance drought tolerance. A summary of studies conducted on drought-stressed plants treated with microalgae, along with their observed effects, is provided in **Table 1**. Additionally, a schematic overview of these findings is presented in **Figure 1**.

**Table 1:** Drought stress mediation in plants by microalgae treatment.

Microalgae species (extract type)	Application method	Plant species	Drought induction	Morphological and physiological changes	Biochemical changes	References
***Chlorella vulgaris*** (methanol extract)	Foliar (1%, 3%, or 5% (v/v))	*Brassica oleracea* var. Italica 'Barokka' (broccoli)	Irrigation deficit to 25% of field capacity	• Shoot length ↑ • Shoot fresh and dry weight ↑ • Leaf area ↑ • Photosynthetic pigment ↑ • RWC ↑ • WUE ↑ • Nutrition uptake ↑	• Membrane damage ↓ • Total flavonoid, phenolic and carotenoids contents ↑ • Enzymatic antioxidant activity ↑	(Kusvuran, 2021)
***Chlorella vulgaris*** (living microalgae)	Foliar (2x10^7^ cfu/mL)	*Cyamopsis tetragonoloba* L. Taub. (guar)	Irrigation deficit to 25%, 50%, 75% and 0% of field capacity	• Shoot height ↑ • Shoot fresh and dry weight ↑ • Root fresh and dry weight ↑ • Leaf number and area ↑ • RWC ↑	• Membrane damage ↓ • Total flavonoid and phenolic contents ↑ • Enzymatic antioxidant activity ↑	(Kusvuran and Kusvuran, 2019)
***Chlorella vulgaris*** (living microalgae or culture supernatant)	Seed and irrigation (1x10^8^ cells/mL)	*Arabidopsis thaliana*	11 days without irrigation and 4 days rehydration	• Fresh weight ↑ • Stomatal closure ↑ • Water content ↑	• Membrane damage ↓ • Total glucosinolate content ↑ • Glucosinolate-related gene expression ↑	(Moon et al., 2024)
***Chlorella saccharophila*** (living microalgae)	Seed and irrigation (5% solution of 2x10^4^ cfu/mL)	*Trigonella foenum-graecum* L. (fenugreek)	Irrigation deficit 50% and 25%	• Ion leakage in leaf tissues ↑	• Membrane damage ↓ • Total antioxidant activity ↑	(Yolci et al., 2022)
***Chlorella saccharophila*** (living microalgae)	Seed and irrigation (5% solution of 2x10^4^ cfu/mL)	*Triticum aestivum* L. cv. Slemani-2 (wheat)	Irrigation deficit 50% and 25% of normal irrigation	• Plant fresh and dry weight ↑ • Root fresh weight ↑ • Photosynthetic pigment ↑ • Nutrition uptake ↑	• Total flavonoid contents ↑	(Salih et al., 2022)
***Chlorella saccharophila*** (living microalgae)	Seed and irrigation (5% solution of 2x10^4^ cfu/mL)	*Glycine max* L. var. Ansoy (soybean)	Irrigation deficit 50% and 25% of normal irrigation	• Shoot length ↑ • Shoot fresh and dry weight ↑ • Root length ↑ • Root fresh and dry weight ↑ • Nutrition uptake ↑	• Total flavonoid and anthocyanin contents ↑	(Oral et al., 2021)
***Chlorella saccharophila*** (living microalgae)	Seed and irrigation (5% solution of 2x10^4^ cfu/mL)	*Calendula officinalis* L. (Aynisafa)	Irrigation deficit 50% and 25% of normal irrigation	• Photosynthetic pigment ↑ • Nutrition uptake ↑	• Membrane damage ↓ • Total flavonoid and phenolic contents ↑ • Total antioxidant activity ↑	(Selem et al., 2022)
***Chlorella sorokiniana*** (methanol extract)	Hydroponics medium (2 mg C_organic_/L)	*Zea mays* P0943, Pioneer Hi-Bred Italia Sementi S. R. L. (maize)	10% PEG6000 medium	• Root length ↑ • Root area and volume ↑ • Lateral root number ↑ • Stomatal conductance ↑	• Efficiency of PSII ↑	(Martini et al., 2021)
***Chlorella sorokiniana*** (living microalgae)	Soil treatment (1x10^6^ cfu/mL on alignate beads)	*Sorghum bicolor* L. Moench cv. Honey Graze	Arid soil	• Soil organic matter ↑ • Shoot dry weight ↑ • Root length ↑ • Root dry weight ↑		(Trejo et al., 2012)
***Arthrospira platensis*** (living microalgae)	Foliar (1% or 2%)	*Citrus reticulata* 'Murcott' (mandarin)	Irrigation deficit 75% and 85% of crop evapotranspiration	• WUE ↑ • Yield quantity ↑ • Yield quality ↑ • Shelf life ↑	• Membrane damage ↓ • Proline levels ↑ • Enzymatic antioxidant activity ↑	(Elmenofy et al., 2023)
***Arthrospira platensis*** (NS)	Foliar (3 g/L)	*Vitis vinifera* L. cv. Pinot Nero	Irrigation deficit 40% of field capacity	• Yield quantity ↑ • Yield quality ↑		(Salvi et al., 2020)
***Arthrospira platensis*** (water extract)	Seed priming (2.5% w/v)	*Triticum aestivum* L. cv. Sakha95 and Shandawel1 (wheat)	22 days without irrigation during heading stage	• Plant height ↑ • Leaf fresh and dry weight ↑ • Leaf area ↑ • Photosynthesis ↑ • Stomatal conductance ↑ • Photosynthetic pigment ↑ • RWC ↑ • WUE ↑ • Yield quantity ↑ • Yield quality ↑	• Carotenoids content ↑ • Sugar levels ↑	(Elnajar et al., 2024)
***Asterarcys quadricellularis*** (suspension of microalgal powder )	Foliar (0.5 mL/L and 1 mL/L (v/v) from 0.25 g/L)	*Phasealus vulgaris* cv. IAC1850 and BRS ESTEIO (common bean)	7 days without irrigation and rehydratation	• Shoots fresh weight ↑ • Leaf area ↑ • Leaf thickness ↑ • Photosynthetic pigment ↑	• Carotenoids content ↑ • Sugar levels ↑ • Protein levels ↑ • Proline levels ↑ • Enzymatic antioxidant activity ↑	(Marques et al., 2023)
***Euglena gracilis*** (extracted β-(1,3)-glucan)	Aeroponics medium (500 mg/L)	*Solanum lycopersicum* L., cv. Micro-tom (tomato)	Delayed time between misting events (from 5 minutes to 120 minutes)	• Vegetative dry weight ↓ • Leaf water potential ↑ • Photosynthesis ↑ • Stomatal conductance ↑ • Yield quantity ↑ • Yield quality ↑	• Efficiency of PSII ↑	(Barsanti et al., 2019)
***Nannochloris* sp. 424-1** (mixture of extracted proteins, osmoprotectants and phytohormones extract)	Foliar (2 mL 0.5% per plant	*Lycopersicum esculentum* cv. Cristal F1 (tomato)	Receiving 2/5 times rewatering every week to FC	• Shoot height ↑ • Root length ↑		(Oancea et al., 2013)

↑, increase compared to control; ↓, decrease compared to control; NS, Not Specified.

### 3.2 Morphological adjustments

When plants encounter water shortages, the resulting yield loss is often the primary concern for farmers. Research on mandarin, tomato, vines, and wheat has demonstrated that the detrimental effects of drought-induced yield losses can significantly be mitigated through the application of microalgae. In addition to offsetting yield losses, these studies have also highlighted improvements in the quality of the harvested products (Barsanti et al., 2019; Salvi et al., 2020; Elmenofy et al., 2023; Elnajar et al., 2024). For example, Elmenofy et al. (2023) observed that applying living *Arthrospira platensis* as a foliar spray twice per season on ‘Murcott’ mandarin (*Citrus reticulata*) prevented yield loss and resulted in a 19-35% increase compared to untreated controls. The treatment enhanced water-use efficiency (kg/m³) beyond that of even well-watered plants. Also qualitative effects were noticed, i.e. mandarins had juicier interiors and increased acidity, along with an extended shelf life. However, in other crop production systems, the potential application of microalgae presents more complex outcomes. In the study by Salvi et al. (2020), foliar treatment of drought-stressed *Vitis vinifera* L. cv. Pinot Nero with *A. platensis* led to an increase in berry weight. While this increase came along with some advantages, such as higher total anthocyanin and polyphenol levels, it also resulted in a lower sugar content and extractable anthocyanins. In addition to its effect on yield, drought also induces various morphological changes, such as reduced plant height and weight, altered leaf morphology (e.g., smaller leaf areas and leaf rolling), and modifications in root structures crucial for water uptake—these include the elongation and thinning of fine roots (Werner et al., 1999; Yang et al., 2021). Oancea et al. (2013) demonstrated that the application of a complex *Nannochloris* sp. 424-1 extract to the leaves of drought-stressed tomato plants mitigated the adverse effects on plant height by 50%. Additionally, the microalgal treatment restored root length to levels comparable to those of well-watered plants. A comparable effect on root length was noted by Martini et al. (2021) in hydroponically-grown maize treated with a methanolic extract of *Chlorella sorokiniana* under 10% PEG6000-induced water stress. The addition of *C. sorokiniana* to its medium not only enhanced root length but also promoted the development of more lateral roots, as well as increased root area and volume. Trejo et al. (2012) provided a proof of concept that *C. sorokiniana* could be utilized to enhance crop production in arid regions. They successfully cultivated *Sorghum bicolor* in arid soils amended with living *C. sorokiniana*, noting a rapid improvement in soil organic matter content and enhanced root and stem development across most growth cycles. A summary of the morphological effects of microalgal treatments on drought-stressed plants is presented in **Figure 1** under the ‘Morphology’ section.

### 3.3 Physiological modifications

Reduced water availability also leads to a diminished influx of nutrients, which can subsequently result in symptoms of nutrient deficiency in plants (Yang et al., 2021). However, seed priming combined with irrigation using living *Chlorella saccharophila* has been shown to significantly enhance root and stem biomass in both wheat and soybean. Additionally, the nitrogen balance index, calculated as the ratio of chlorophyll to flavonoids in leaves and serving as an indicator of plant nutritional status regarding nitrogen levels, was notably higher in plants treated with microalgae (Oral et al., 2021; Salih et al., 2022). These observations suggest that *C. saccharophila* plays a pivotal role in enhancing nutrient uptake under drought conditions. The same research group further substantiated these findings in *Calendula officinalis* L., where a similar increase in nutrient uptake was observed under water-stressed conditions following treatment with *C. saccharophila* (Selem et al., 2022).

The aforementioned morphological changes are frequently accompanied by physiological alterations induced by drought. Photosynthesis, a critical physiological process, is particularly vulnerable to reductions in soil water content (Dinh et al., 2019). The decline in photosynthetic activity under drought conditions can be attributed to both stomatal and non-stomatal limitations, depending on the severity of the water deficit (Yang et al., 2021). Mild drought typically results in decreases in stomatal conductance, which restrict CO_2_ uptake and transpiration. Severe drought has also a strong impact on non-stomatal factors, including reduced enzyme activity and limited availability of essential photosynthetic components, such as ribulose-1,5-bisphosphate carboxylase/oxygenase and ribulose-1,5-bisphosphate (Deeba et al., 2012). Stomatal responses, including closure and developmental changes such as increased stomatal length and width coupled with decreased density, are among the most known mechanisms to conserve water (Martin-StPaul et al., 2017; Zhao et al., 2020; Yang et al., 2021). In Li et al. (2014) demonstrated that foliar application of *C. vulgaris* to *Vicia faba* induces partial stomatal closure via NADPH oxidase-dependent reactive oxygen species (ROS) production, thereby enhancing water use efficiency (WUE). This effect was observed with both live and heat-killed *C. vulgaris*, suggesting that the components responsible for the observed benefits are heat-resistant. These findings may offer a mechanistic basis for the increased relative water content (RWC) observed in drought-stressed guar (*Cyamopsis tetragonoloba* L. Taub.) seedlings following foliar application of living *C. vulgaris* (Kusvuran and Kusvuran, 2019). Contrary to these findings, other studies have not reported increased water retention in microalgae-treated plants due to stomatal closure. For example, Barsanti et al. (2019) found that water-stressed tomato plants treated with β-(1,3)-glucan extracted from *Euglena gracilis* in an aeroponic medium exhibited improved leaf water potential, stomatal conductance and internal CO_2_ concentrations compared to water-stressed controls and were similar to those of well-watered plants. Furthermore, the photochemical efficiency, actual photon yield, and quenching state of photosystem II (PSII), which are typically diminished under drought stress, were comparable to those of well-watered controls. This led to an enhanced photosynthetic rate and increased sugar content in the tomato fruits. A similar phenomenon was observed by Elnajar et al. (2024), who reported improved WUE and RWC in wheat plants whose seeds had been primed with a water extract of *A. platensis* under drought conditions. In this study, the transpiration rate remained unchanged compared to water-stressed controls, while changes in stomatal conductance varied depending on the cultivar. Nevertheless, microalgae-treated wheat plants exhibited elevated levels of photosynthetic pigments and an enhanced photosynthetic rate, resulting in higher sugar content compared to untreated plants under drought stress. Photosynthetic pigments such as chlorophyll and carotenoids, which are crucial for photosynthesis, generally decrease under drought conditions (Farooq et al., 2009). Several studies have reported increases in these pigments in response to microalgal treatments. For instance, enhanced levels of chlorophyll and carotenoids were observed in common bean sprayed with *Asterarcys quadricellularis* (Marques et al., 2023), in broccoli treated with foliar applications of a methanolic extract of *C. vulgaris* (Kusvuran, 2021), and in wheat and *Calendula officinalis* subjected to seed priming and irrigation with living *C. saccharophila* (Salih et al., 2022; Selem et al., 2022). In these cases, the increase in photosynthetic pigments was often correlated with improved nutrient uptake. This enhanced nutrient uptake may account for the rise in photosynthetic pigments, as the continuous metabolism of these pigments in plants is typically downregulated under drought-stress signal transduction and nutrient deficiency (Yang et al., 2021).

### 3.4 Biochemical changes

#### 3.4.1 Osmotic regulation

Osmotic regulation is a critical adaptive mechanism in plants for mitigating water stress, primarily achieved through pathways such as the reduction of intracellular water and cell volume, along with the accumulation of compatible cellular solutes. This process helps to maintain turgor pressure, stomatal function, and key biochemical activities needed for plant growth and photosynthesis during drought (Yang et al., 2021). Both organic and inorganic osmolytes play significant roles in this regulation, with organic compounds such as glucosinolate and proline contributing to cellular stability and protection against oxidative damage (Wang et al., 2004a). In a study investigating the mode of action of *C. vulgaris* culture supernatant in *A. thaliana*, irrigation with this supernatant not only induced stomatal closure but also increased glucosinolate content. This finding was further substantiated by targeted real-time quantitative polymerase chain reaction, which revealed enhanced expression of the transcription factors *MYB28* and *MYB29*, both known to positively regulate glucosinolate biosynthesis (Moon et al., 2024). Similarly, another osmolyte, proline, showed significant accumulation under drought conditions, with increases of 50% in mandarin peels and 12% in common bean leaves following foliar treatments with living *A. platensis* and *A. quadricellularis* powder suspension, respectively (Elmenofy et al., 2023; Marques et al., 2023). In addition to elevated proline levels, the study by Marques et al. (2023) reported an increase in total sugar content in common bean leaves treated with *A. quadricellularis.* This finding underscores the role of sugars in osmotic regulation, which, as previously reported by Silva et al. (2010) and Gurrieri et al. (2020), may even surpass the importance of proline under conditions of mild stress. Furthermore, Marques et al. (2023) observed a 36% increase in total protein content. Although the specific proteins involved were not identified, it can be hypothesized that these proteins may include key drought-induced osmotic regulatory proteins such as Late embryogenesis abundant (LEA) proteins, dehydrin, and aquaporin. LEA proteins, rich in lysine and glycine, are known to maintain cellular hydration and scavenge ROS (Soulages et al., 2003; Hara et al., 2004). Dehydrins stabilize cellular membranes and prevent protein denaturation (Allagulova et al., 2003), while aquaporins facilitate water transport across membranes, crucial for regulating turgor pressure and maintaining cell integrity (Netting, 2000). Despite their importance, the levels of these proteins have not systematically been quantified in plant drought studies with or without microalgae addition to date. However, a transcriptome analysis of well-watered tomato plants treated with a cell suspension of *Chlorella* sp. MACC-360 via the soil drench method revealed upregulation of LEA and dehydrin genes (TAS14, embryogenic cell protein 40 and dehydrin), along with genes related to the biosynthesis of cutin, suberin, and wax, suggesting a preventive response to potential drought (Gitau et al., 2023).

#### 3.4.2 Oxidative stress

ROS are central to the cellular damage associated with water deficit. While they are produced in plants as by-products of normal metabolic processes, including those occurring in mitochondria, chloroplasts, peroxisomes, and plasma membranes (Mignolet-Spruyt et al., 2016), their levels can surpass the plant’s scavenging capacity under drought conditions, leading to oxidative stress. This oxidative stress can cause damage to cellular membranes, proteins, and DNA. Nevertheless, ROS also play a role in plant defense mechanisms and growth regulation. Plants have evolved both enzymatic (e.g., superoxide dismutase (SOD), catalase (CAT), ascorbate peroxidase (APX)) and non-enzymatic (e.g., ascorbate, glutathione) antioxidant systems to mitigate ROS damage and maintain homeostasis under stress (Nadarajah, 2020). Improved antioxidant activity, both enzymatic and non-enzymatic, appears to be a significant mechanism by which microalgae assist plants in coping with drought stress. This effect has been documented in more than half of the current studies on drought stress alleviation using microalgae. Yolci et al. (2022) demonstrated that living *C. saccharophila*, used as a seed primer and irrigation supplement for fenugreek (*Trigonella foenum-graecum* L.) under a 50% irrigation deficit, enhanced total antioxidant activity. This increase in antioxidant activity was accompanied by reduced membrane damage, as evidenced by a decrease in malondialdehyde (MDA) levels. MDA, a product of lipid peroxidation in plant membranes, is commonly used as an indicator of oxidative stress and membrane damage. However, ion leakage increased simultaneously, indicating compromised cell membrane integrity, which contrasts with the earlier observations. Kusvuran and Kusvuran (2019) reported similar findings, where living *C. vulgaris* foliar treatment of drought-stressed guar plants significantly enhanced the activity of SOD, CAT, APX, and glutathione reductase, along with increasing non-enzymatic antioxidants like flavonoids and phenolic compounds, leading to reduced MDA levels and less membrane damage.

#### 3.4.3 Hormonal signaling

Drought-induced morphological, physiological, and biochemical changes are often linked to alterations in hormonal synthesis and distribution. Phytohormones such as abscisic acid (ABA), auxin, cytokinin, and ethylene play essential roles in modulating drought responses, influencing various processes ranging from root architecture to osmotic balance. Among these, ABA is particularly notable for its well-documented role in inducing drought tolerance. ABA is crucial for regulating stomatal closure, stimulating the expression of genes related to LEA proteins and dehydrins, and promoting the accumulation of osmoprotectants (Wahab et al., 2022). Although many studies mention potential hormonal effects of microalgal treatments on drought stress resilience, few actual hormone levels have been measured in plants. This is likely due to the complexity and high costs associated with hormone analysis. It is well-established that microalgae contain phytohormones or phytohormone-like compounds. For example, *Chlorella* spp. are known to contain significant levels of ABA, cytokinins, auxins, and 1-aminocyclopropane-1-carboxylic acid (ACC) (Kapoore et al., 2021). In this context, *C. vulgaris* was shown to preemptively upregulate genes related to auxin biosynthesis and transduction pathways in lettuce, while in tomato, it upregulated ethylene- and ABA-related genes (Gitau et al., 2023; Santoro et al., 2023). Notably, the response to microalgal treatment appears to be species-specific, as demonstrated by Santoro et al. (2023) where a methanolic extract of *Scenedesmus quadricauda* was found to upregulate genes related to cytokinin biosynthesis pathways in lettuce, contrasting with the auxin-related gene upregulation observed with *C. vulgaris*. Although research on hormonal changes in plants induced by microalgal treatment remains limited, the potential applications in biotechnology are significant. For instance, Yuan et al. (2014) demonstrated that transgenic *A. thaliana* plants expressing IgASE1, a C18-Δ9-specific polyunsaturated fatty acid elongase from *Isochrysis galbana*, exhibited enhanced drought tolerance. These transgenic plants showed increased sensitivity to ABA, and under simulated drought conditions using 300 mM mannitol, there was an upregulation of genes involved in ABA biosynthesis and other stress-related pathways.

In conclusion, microalgal biostimulants offer promising solutions to mitigate drought stress in plants, addressing challenges at morphological, physiological, and biochemical levels. Their effects include improved water-use efficiency, enhanced nutrient uptake, and alleviation of oxidative stress, often through mechanisms such as osmotic regulation and hormonal modulation (**Figure 1**). While studies consistently report benefits such as increased yield, improved product quality, and resilience against drought-induced damage, the underlying mechanisms remain partially understood, particularly in terms of genetic, proteomic, and hormonal pathways.

## 4 Turning the tide: microalgal solutions for salt-stressed crops

### 4.1 Introduction

Salinity is a major abiotic factor that impairs plant growth, development, and productivity, especially in arid and semi-arid regions. The increasing salinity levels are attributed to various factors such as poor irrigation practices, improper use of fertilizers, deforestation, and climate change (Zhao et al., 2021; FAO, 2022; Fu and Yang, 2023). Approximately 10% of the world’s total land area and 50% of irrigated agricultural lands are affected by salinity, leading to decreased yields that cost the agricultural sector an estimated $12–27 billion annually (Behera et al., 2022; FAO, 2022). Projections indicate that salinity issues are only expected to rise in the future. This will undoubtedly accelerate the development of sustainable solutions like microalgal biostimulants to improve crop resilience (Yang and Guo, 2018; FAO, 2022). **Table 2** provides a summary of microalgal treatments applied to salt-stressed plants and their observed effects. Additionally, **Figure 1** presents a schematic overview of these findings.

**Table 2:** Salt stress mediation in plants by microalgae treatment.

Microalgae species (extract type)	Application method	Plant species	Salinity induction	Morphological and physiological changes	Biochemical changes	References
***Chlorella ellipsoida*** (water extract)	Irrigation (5 g/L)	*Triticum aestivum* L. cv. Giz94 (Wheat)	10% and 20% seawater irrigation		• Total protein content ↑ • Antioxidant activity ↑	(El-Baky et al., 2010)
***Chlorella pyrenoidosa*** (living microalgae)	Seed priming and irrigation (2.21*10^10^ cell/L as 25%; 50%, 75% and 100% algal solution)	*Chenopodium quinoa*	Germination: 100, 200, and 300 mM NaCl solutions and Irrigation: natural saline soils	• Germination ↑ • Root and shoot length ↑ • Root branch number ↑ • Plant fresh weight ↑ • Leaf length and width ↑ • Nutrition uptake ↑		(Ma et al., 2022)
***Chlorella vulgaris*** (living microalgae)	Foliar (2x10^7^ cells/mL)	*Cyamopsis tetragonoloba* L. Taub. (guar)	100 mM NaCl irrigation at day 39	• Shoot length ↑ • Number of stems ↑ • Shoot fresh and dry weight ↑ • Leaf number and area ↑ • Photosynthetic pigment ↑	• Membrane damage ↓ • Enzymatic antioxidant activity ↑ • Total flavonoid, phenolic and carotenoids contents ↑ • Ca^2+^ and K^+^ content ↑ • Na^+^ and Cl^-^ content ↓	(Kusvuran and Can, 2020)
***Chlorella vulgaris*** (living microalgae)	Irrigation (1.5*10^7^ cells/mL)	*Moringa oleifera*	3000 and 6000 ppm seawater irrigation (3 and 6 g/L salt)	• Plant height ↑ • Stem diameter ↑ • Stem, leaves and root dry weight ↑ • Photosynthetic pigment ↑ • Yield quantity ↑ • Yield quality ↑	• Total carotenoids contents ↑ • K^+^ content ↑ • Na^+^ content ↓	(Al Dayel and El Sherif, 2021)
***Chlorella vulgaris*** (water extract)	Seed priming (10% w/v), irrigation and foliar (1% w/v)	*Solanum lycopersicum* cv. Agyad (tomato)	2,4 and 7 dSm/L saline water irrigation	• Plant height ↑ • Leaf area ↑ • Fresh and dry weight ↑ • Yield quantity ↑ • Yield quality ↑ • Shelf life ↑		(Mostafa et al., 2023)
***Chlorella vulgaris*** (living microalgae)	Seed (1x10^6^ cells/mL on alignate beads)	*Lycopersicum esculentum* var. Cherry (tomato)	50, 10, 150, 200 and 250 mM NaCl hydroponics medium	• Stem length ↑		(Escalante et al., 2015)
***Chlorella vulgaris*** (extracted oligosaccharides)	Seed (25, 37.5, 75 and 150 µg/L)	*Oryza sativa* (rice)	3, 6, 9 and 12 g/L NaCl solution	• Germination ↑ • Water accumulation ↑ • Nutrition uptake ↑	• Membrane damage ↓ • Proline levels ↑ • Enzymatic antioxidant activity ↑ • Sugar levels ↑	(Wang et al., 2024)
***Chlorella* sp.** (water extract)	Irrigation (1, 3, 5 mL extract/L of 50 g/L stock)	*Triticum aestivum* L. (wheat)	100 mM NaCl irrigation	• Seedling length ↑ • Seedling and root fresh weight ↑	• Efficiency of PSII ↑ • Membrane damage ↓ • Proline levels ↓ • Enzymatic antioxidant activity ↓ • Salicylic acid, ABA, auxin and gibberellic acid content ↑ • Jasmonic acid and cytokinin content ↓	(Liu et al., 2024)
***Arthrospira platensis*** (NS)	Foliar (0.1 g/L)	*Vicia faba* cv. Giza 2 (broad bean)	135 mM NaCl (13 dS/m) irrigation	• Photosynthesis ↑ • Transpiration rate ↑ • Photosynthetic pigment ↑ • Nutrition uptake ↑ • Yield quantity ↑	• Membrane damage ↓ • Proline levels ↓ • Enzymatic antioxidant activity ↓ • Total phenolic and carotenoids contents ↑ • Total protein content ↑ • K^+^ content ↑ • Na^+^ and Cl^-^ content ↓	(Selem, 2019)
***Arthrospira platensis*** (water extract)	Seed priming (10% w/v), irrigation and foliar (1% w/v)	*Solanum lycopersicum* cv. Agyad (tomato)	2, 4 and 7 dSm/L saline water irrigation	• Plant height ↑ • Leaf area ↑ • Fresh and dry weight ↑ • Yield quantity ↑ • Yield quality ↑ • Shelf life ↑		(Mostafa et al., 2023)
***Arthrospira platensis*** (hydrolysate)	Foliar (5 g/L)	*Pelargonium hortorum* L.H. (Bailey)	2.0, 3.0, and 3.5 dS/m NaCl irrigation	• Number of flowers ↑		(Tejada-Ruiz et al., 2020)
***Arthrospira platensis*** (hydrolysate)	Foliar (5 g/L)	*Petunia x hybrida* cv. Surfinia Purple	2.0, 3.0, and 3.5 dS/m NaCl irrigation	• Shoot number ↑ • Flower number ↑ • Leaf length and number ↑ • Root, flowers and total dry weight ↑		(Bayona-Morcillo et al., 2020)
***Arthrospira platensis*** (water extract)	Irrigation (2% liquid extract)	*Triticum aestivum* L. (wheat)	10% and 25% seawater irrigation	• Shoot and root length ↑ • Leaf area and number ↑ • Total fresh and dry weight ↑	• Total antioxidant activity ↑ • Total sugar content ↑ • Total protein content ↑	(Hamouda et al., 2022)
***Arthrospira platensis*** (water extract)	Seed priming (0, 5, 10 and 15 ml/L of 150 g/L stock)	*Calotropis procera* var. Aiton (milkweed)	0, 7.5, 15 and 30 dS/m seawater irrigation	• Germination ↑ • Shoot and root length ↑ • Root dry weight ↑		(Bahmani Jafarlou et al., 2021)
***Dunaliella salina*** (hydrolysate or extracted exopolysaccharide)	Seed (hydrolysate: 0.01% of dry weight; exopoly-saccharides: 2 mg/L)	*Triticum aestivum* cv. Amal (wheat)	3 and 6 g/L NaCl solution	• Germination ↑ • Shoot and root length ↑	• Proline levels ↓ • Enzymatic antioxidant activity ↓	(El Arroussi et al., 2016)
***Dunaliella salina*** (extracted carbohydrates)	Seed (conc. adjusted to 3.4 mg carbohydrates/mL)	*Capsicum annuum* L. (bell pepper)	25 and 50 mM NaCl solution	• Root length ↑ • Shoot and root fresh weight ↑	• Membrane damage ↓ • ROS production ↓ • Enzymatic antioxidant activity dependent on stress severity	(Guzmán-Murillo et al., 2013)
***Dunaliella salina*** (extracted exopolysaccharide)	Foliar (0.1 g/L)	*Solanum lycopersicum* var. JANA F1 (tomato)	3 and 6 g/L NaCl soil mixture	• Shoot height ↑ • Shoot and root dry weight ↑ • Photosynthetic pigment ↑	• Proline levels ↓ • Enzymatic antioxidant activity ↓ • Total phenolic contents ↑ • K^+^ content ↑ • Fatty acid content ↓ • 2,4-di-tetr-butylphenol ↑ • Tocopherol ↓	(El Arroussi et al., 2018)
***Dunaliella salina, Chlorella ellipsoidea, Aphanothece* sp*; and Arthrospira maxima* consortium** (sulfuric acid extract)	Irrigation (1, 5 and 10% v/v)	*Solanum lycopersicum* var. JANA F1 (tomato)	80, 120, and 150 mM NaCl irrigation	• Shoot height ↑ • Shoot and root fresh weight ↑ • Photosynthetic pigment ↑ • Nutrient uptake ↑	• Membrane damage ↓ • Proline levels ↑ • Enzymatic antioxidant activity ↑ • Total carotenoids contents ↑ • K^+^/Na^+^ ratio ↑ • Alkanes ↑ • Fatty acid content ↓	(Mutale-joan et al., 2021)
***Klebsormidium* sp.** (water extract)	Seed (2 mL supernatans of 1 month old culture)	*Arabidopsis thaliana* (Col-10)	125 mM NaCl solution	• Root length ↑ • Leaf number ↓		(Roque et al., 2023)
***Klebsormidium* sp.** (water extract)	Seed (2 mL supernatans of 1 month old culture)	*Lolium multiflorum* (cv. Diamond T.)	100 mM NaCl solution	• Fresh weight ↑		(Roque et al., 2023)
***Nannochloropsis salina*** (living microalgae)	Irrigation (1.5*10^7^ cells/mL)	*Moringa oleifera*	3000 and 6000 ppm seawater irrigation (3 and 6 g/L salt)	• Plant height ↑ • Stem diameter ↑ • Stem, leaves and root dry weight ↑ • Photosynthetic pigment ↑ • Yield quantity ↑ • Yield quality ↑	• Total antioxidant activity ↑ • Total sugar content ↑ • Total protein content ↑	(Al Dayel and El Sherif, 2021)
***Phaeodactylum tricornutum*** (extracted carbohydrates)	Seed (conc. adjusted to 3.4 mg carbohydrates/mL)	*Capsicum annuum* L. (bell pepper)	25 and 50 mM NaCl solution	• Root length ↑ • Shoot and root fresh weight ↑	• Membrane damage ↓ • ROS production ↓ • Enzymatic antioxidant activity dependent on stress severity	(Guzmán-Murillo et al., 2013)

↑, increase compared to control; ↓, decrease compared to control; NS, Not Specified.

Salt stress refers to the adverse effects on plants caused by high concentrations of soluble salts in the soil or water. Among these salts, sodium chloride (NaCl) is the primary contributor, while other salts, such as sodium sulfate (Na_2_SO_4_), play a comparatively minor role. Salt stress is caused by both osmotic and ionic stress. Osmotic stress reduces the soil’s water potential, making it difficult for plants to absorb water and nutrients, thereby creating drought-like conditions even when moisture is present. Ionic stress arises from the excessive uptake of sodium (Na^+^) and chloride (Cl^−^) ions, disrupting the ionic balance within plant cells. These ions can accumulate to toxic levels in plant tissues, interfering with metabolic functions and causing cellular damage (Fu and Yang, 2023). Both osmotic and ionic stresses can induce secondary stresses such as oxidative stress due to the production of ROS, which further damage cellular structures. The severity of salt stress plays a significant role in determining plant responses (Fu and Yang, 2023). Mild salt stress, often associated with electrical conductivity levels of 2–4 dS/m, may actually improve certain traits in some crops, such as increased antioxidant content or enhanced fruit quality in species like strawberry and tomato (Galli et al., 2016). However, in salt-sensitive plants such as rice and maize, even mild stresses can have adverse effects (Munns, 2002; De Azevedo Neto et al., 2006). In maize, for instance, salinity levels of 2.5 dS/m resulted in a yield reduction of approximately 10%, while levels of 5.5 dS/m caused a 50% decrease in yield (Panta et al., 2014). Moderate salt stress—corresponding to electrical conductivity levels of 5–8 dS/m or NaCl concentrations between 50 and 150 mM—significantly affects photosynthesis and disrupts ion balance in plants. However, (partial) recovery is possible if the stress is alleviated (Claeys et al., 2014; Ma et al., 2017). Severe salt stress, exceeding 8 dS/m or 150 mM NaCl, strongly inhibits growth and can lead to plant mortality (Yang and Guo, 2018). To mitigate these adverse effects, microalgae can act directly as biostimulants on plants or indirectly by contributing to the desalination of soils or water. Certain microalgal species have demonstrated the ability to desalinize water or soil through adsorption and absorption mechanisms. For example, living *Scenedesmus obliquus* was shown to reduce the NaCl content in brackish water (6800–8800 ppm) by 20% within just 30 minutes of contact, primarily through adsorption and the formation of a singular molecular layer around its cells (Wei et al., 2020). The freshwater algae *C. vulgaris* exhibited even greater efficiency, reducing the electrical conductivity of brackish water by 80% and 40% at NaCl concentrations of 1000 ppm and 5000 ppm, respectively (Barahoei et al., 2021). At even higher salinity levels (130 mS/cm or ~71500 ppm), the marine microalga *D. salina* was effective in desalinating water, reducing salinity by 40–45 mS/cm over seven days through absorption mechanisms (Moayedi et al., 2019). This indirect effect of microalgal application reduces the impact of salt stress on plants, thereby enhancing their resilience to stress. While not traditionally classified as such, it can be argued that this indirect effect also qualifies as a biostimulant action, as it contributes to increasing plant tolerance to abiotic stress, one of the primary functions of biostimulants.

### 4.2 Morphological adjustments

The potential of microalgae as biostimulants to directly mitigate salt stress in plants should not be overlooked. Broad bean (*Vicia faba* cv. Giza 2) irrigated with highly saline water (13 dS/m) exhibited a 22% reduction in yield, measured as the weight of 100 seeds. However, a single foliar application of *A. platensis* at the flowering stage significantly mitigated the yield loss, reducing it to just 4% compared to unstressed plants (Selem, 2019). In addition to its effects on food crops, *A. platensis* has also demonstrated efficacy in ornamental plants. When applied as a hydrolysate foliar treatment (5 g/L), it increased flower production in *Petunia x hybrida* and bailey (*Pelargonium hortorum*) (Bayona-Morcillo et al., 2020; Tejada-Ruiz et al., 2020). Notably, in bailey, this treatment not only alleviated the 30% decline in flower numbers caused by salt stress but also enhanced flowering beyond the levels observed in unstressed control plants (Tejada-Ruiz et al., 2020). In addition to *A. platensis*, other microalgae such as *C. vulgaris* and *N. salina* have proven to be effective in ameliorating the effects of salt stress on plant yield. Irrigation with these living microalgae increased the harvestable parts (leaves) of *Moringa oleifera*, a crop cultivated for medicinal and food uses in Saudi Arabia, under salt stress conditions. Furthermore, the treatment enhanced crop quality by increasing the concentrations of rutin and gallic acid in the leaves, two bioactive compounds with significant pharmaceutical applications. At the highest salinity level tested (6 g/L NaCl), untreated *Moringa oleifera* plants did not survive. In contrast, plants treated with microalgae not only survived but also maintained the same number of leaves as the unstressed control, albeit with reduced leaf quality compared to the control plants (Al Dayel and El Sherif, 2021). In addition to improving harvestable yield, Al Dayel and El Sherif (2021) reported increased root and stem biomass in microalgae-treated plants compared to salt-stressed controls. Similar enhancements in overall growth have been reported in other species, including tomato, wheat, bell pepper, *A. thaliana*, *Petunia x hybrida*, milkweed, and quinoa, suggesting a broad potential for microalgae-based biostimulants in alleviating salt stress-induced growth reduction across a diverse range of crops (Guzmán-Murillo et al., 2013; El Arroussi et al., 2016, 2018; Bayona-Morcillo et al., 2020; Bahmani Jafarlou et al., 2021; Ma et al., 2022; Roque et al., 2023). Root growth is of particular interest as roots serve as the primary interface between plants and saline soils. Salt stress adversely affects root development, reducing root mass and altering root architecture by inhibiting lateral root formation. Shoot biomass is also reduced, albeit to a lesser extent than roots, resulting in a lower root-to-shoot ratio. Leaves are similarly affected, with reduced expansion, decreased leaf area, and accelerated senescence (Negrão et al., 2017; Fu and Yang, 2023). Such effects were observed in tomato plants irrigated with 7 dS/m saline water, but the addition of either *C. vulgaris* or *A. platensis* water extracts as a combined seed priming, irrigation, and foliar treatment mitigated these adverse effects. The treatment enhanced leaf area, plant height, and biomass, ultimately improving yield compared to salt-stressed controls (Mostafa et al., 2023). Similar benefits were observed in milkweed (*Calotropis procera* Aiton), where seed priming with an *A. platensis* water extract improved root growth and increased the root-to-shoot ratio (Bahmani Jafarlou et al., 2021). In *Chenopodium quinoa*, Ma et al. (2022) observed comparable results with an irrigation of *Chlorella pyrenoidosa*. Plants treated with this living microalgae displayed improved growth characteristics, including increased root length, lateral root formation, shoot length, biomass, root-to-shoot ratio, and expanded leaf dimensions (length and width). These enhancements were achieved on natural saline soil, highlighting the potential of *C. pyrenoidosa* in real world practices.

Osmotic stress induced by salinity reduces the soil’s water potential, thereby limiting water availability to plants. This condition disrupts numerous physiological and biochemical processes essential for plant development. Germination, for instance, is particularly sensitive to osmotic stress, as water must diffuse into the seed coat to initiate the process (De La Reguera et al., 2020). Microalgal biostimulants have demonstrated the capacity to alleviate the negative effects of salinity on germination across a wide range of crops, from ornamentals such as milkweed to high-yield species such as tomato (Bahmani Jafarlou et al., 2021; Mostafa et al., 2023). A notable example is provided by Wang et al. (2024), who studied the germination of rice seeds in saline solutions (NaCl) with or without the addition of *C. vulgaris* oligosaccharides. Their results revealed that microalgal treatment increased water uptake in rice seeds by approximately 5–15%, significantly enhancing germination rates for all tested salt concentrations (3–12 g/L) (Wang et al., 2024). The morphological effects of microalgal treatments on salt-stressed plants are outlined in **Figure 1** within the ‘Morphology’ section.

### 4.3 Physiological modifications and biochemical changes

#### 4.3.1 The dual challenge: osmotic and ionic stress

The combined osmotic and ionic stresses induced by salinity have profound biochemical effects on plants. Ionic homeostasis, which is closely related to ion transport, is disrupted by elevated Na^+^ concentrations in soil or water. Maintaining a proper potassium-to-sodium (K^+^/Na^+^) ratio is critical for plants to adapt to salt stress, as this balance prevents cellular damage and nutrient deficiencies. Concurrently, osmotic stress caused by salinity reduces water uptake, which inevitably decreases nutrient absorption, further impacting plant growth. A major consequence of salt stress is reduced photosynthesis, caused by lower PSII activity and impaired chlorophyll production. Salt stress also reduces stomatal density and induces stomatal closure, limiting CO_2_ uptake and photosynthesis (Fu and Yang, 2023). Calcium (Ca²^+^) plays a pivotal role in sensing and activating salt stress tolerance responses through the salt overly sensitive (SOS) pathway. This pathway primarily regulates Na^+^ efflux and K^+^ uptake, but it also influences other protective mechanisms, such as stomatal closure, osmoprotectant accumulation, phytohormone signaling, and ROS production (Bachani et al., 2022). A study by Selem (2019) demonstrated a 21% increase in broad bean yield under 135 mM NaCl irrigation following a single foliar application of *A. platensis*. The treatment was linked to enhanced nutrient uptake, including potassium, nitrogen, and phosphorus, alongside improved photosynthetic activity, pigment levels, and transpiration rates. Interestingly, while proline levels—a key osmoprotectant in mediating osmotic stress—were lower in treated stressed plants compared to untreated stressed plants, the concurrent reduction in Na^+^ and Cl^−^ content suggests that the primary mechanism of stress alleviation was ionic stress regulation rather than osmotic stress mitigation. These findings point to the SOS pathway as a likely mechanism, given its critical role in maintaining ionic homeostasis through Na^+^ efflux and K^+^ uptake. Similar findings were reported by Liu et al. (2024), where *Chlorella* sp. water extract irrigation improved the performance index of PSII in wheat plants subjected to 100 mM NaCl stress whilst proline levels were reduced (Liu et al., 2024). In guar plants exposed to 100 mM NaCl, foliar treatment with living *C. vulgaris* resulted in a reduction of Na^+^ and Cl^−^ contents concomitant with an increase of K^+^ and Ca²^+^ levels, further implicating SOS pathway activation (Kusvuran and Can, 2020). However, contrasting results were observed in rice treated with *C. vulgaris* oligosaccharides under salt stress, where increased nitrogen uptake and biomass accumulation were associated with elevated proline and soluble sugar levels, acting as osmoprotectants (Wang et al., 2024). Similarly, Hamouda et al. (2022) reported that wheat plants irrigated with *A. platensis* water extract under salt stress exhibited increased biomass and leaf area, as well as elevated carbohydrate and protein levels. These findings suggest a dual role for microalgal treatments in alleviating both osmotic and ionic stress, depending on the plant species, stress conditions, and the type of microalgal biostimulant.

#### 4.3.2 Oxidative stress

Like many other abiotic stresses, salt stress also induces rapid production of ROS, leading to secondary oxidative damage at the cellular level. As discussed in the section on drought stress, plants activate defensive mechanisms to mitigate ROS accumulation. These include the production of enzymatic antioxidants such as SOD, CAT, and APX, as well as non-enzymatic antioxidants like tocopherol and ascorbate (Fu and Yang, 2023). Similar to osmotic and ionic stress alleviation, microalgal effects on enzymatic and non-enzymatic antioxidant systems under salt stress remain highly variable and context-dependent. El-Baky et al. (2010) first reported that irrigation with *Chlorella ellipsoida* water extract in salt-stressed wheat increased the levels of carotenoids, tocopherol, total phenols, and overall antioxidant activity. Similarly, living *C. vulgaris* foliar application in salt-stressed guar plants resulted in elevated antioxidant activity, including significant increases in SOD (113%), CAT (256%), and APX (56%) enzyme activity. This improvement corresponded with reduced MDA levels, suggesting enhanced cell membrane integrity due to lower lipid peroxidation and oxidative stress (Kusvuran and Can, 2020). In contrast, different results were reported in wheat seedlings treated with *D. salina* exopolysaccharides under salt stress. In this study, the addition of microalgae reduced the activity of ROS-scavenging enzymes, including peroxidase and SOD. This reduction was attributed to a lower perceived stress level in wheat plants, as evidenced by increased root and shoot growth compared to untreated controls (El Arroussi et al., 2016). The apparent variability in antioxidant responses may be influenced by factors such as stress severity and microalgal species. For example, Guzmán-Murillo et al. (2013) demonstrated that the effect of carbohydrates extracted from *D. salina* and *Phaeodactylum tricornutum* on antioxidant activity in salt-stressed bell pepper plants was highly dependent on the salt concentration. At low salinity levels (25 mM NaCl), reductions in SOD and glutathione peroxidase (GPX) activity, along with decreased superoxide radical (O_2_^−^) production, were observed. In contrast, at higher salinity levels (50 mM NaCl), CAT and GPX activity increased. Notably, at both salinity levels, lipid peroxidation was significantly reduced (by 30–50%) compared to untreated controls. An alternative approach to leveraging microalgae for improving salt tolerance by influencing antioxidant mechanisms was demonstrated by Qu et al. (2021). In their study, a novel bZIP transcription factor (*ChbZIP1*) was identified in *Chlorella* sp. BLD and overexpressed in transgenic *A. thaliana*. These transgenic plants exhibited upregulated expression of key genes associated with antioxidant activity, including *GPX1*, *DOX1*, *CAT2*, and *EMB*. Consequently, they displayed elevated levels of antioxidant enzymes such as APX, CAT, and peroxidase, along with reduced MDA content and lower ROS production. These effects translated into longer roots and greater fresh weight compared to wild-type plants under salt stress conditions (Qu et al., 2021).

#### 4.3.3 Lipidomics and hormone signaling

Although the literature remains divided on the effects of microalgal treatments on osmoprotectants, ROS-scavenging enzymes, and non-enzymatic antioxidants under salt stress, two lipidomics studies on tomato (*Solanum lycopersicum* var. JANA F1) reached similar conclusions. One study examined tomatoes grown on saline soil treated with a *D. salina* exopolysaccharide leaf spray (El Arroussi et al., 2018), while the other investigated plants irrigated with saline water supplemented with a sulfuric acid extract from a consortium of *D. salina*, *C. ellipsoidea*, *Aphanothece* sp., and *A. maxima* (Mutale-joan et al., 2021). Despite contrasting effects on osmoprotectants, ROS-scavenging enzymes, and non-enzymatic antioxidant levels, both studies observed increased potassium uptake and reduced sodium uptake, leading to an improved K^+^/Na^+^ ratio. Additionally, both studies reported a decrease in (un)saturated fatty acids and very long-chain fatty acids alongside an increase in alkanes, particularly very long-chain alkanes (El Arroussi et al., 2018; Mutale-joan et al., 2021). Very long chain fatty acids, essential precursors for cuticular waxes and cutin, are converted into very long-chain alkanes, which are highly hydrophobic saturated hydrocarbons contributing to the water-repellent properties of the plant cuticle (Arya et al., 2021). The observed reduction in unsaturated fatty acids is consistent with membrane remodeling processes that increase rigidity, stabilize the membrane under osmotic stress, limit passive Na^+^ influx, and protect lipids from oxidative damage (Gogna et al., 2020). Both studies also noted decreased levels of azelaic acid (C9:0) and α-linolenic acid (C18:3). Azelaic acid primes plants to accumulate salicylic acid, a key defense hormone involved in biotic and abiotic stress responses, while α-linolenic acid serves as a precursor in 13-hydroperoxide biosynthesis, contributing to jasmonic acid synthesis. Jasmonic acid plays a critical role in activating plant tolerance mechanisms under salt stress (Fu and Yang, 2023). El Arroussi et al. (2018) hypothesized that the reduction in both lipids observed in microalgae-treated tomato plants was attributed to a reduced perception of salt stress, leading to lower levels of salicylic acid and jasmonic acid. This hypothesis was further supported by a concomitant decrease in tocopherol levels, an antioxidant whose synthesis is regulated by environmental stress and stress-related phytohormones such as jasmonic acid and salicylic acid. Additionally, reductions in proline levels and enzymatic antioxidants, including CAT and SOD, were observed. However, the precise mechanism through which these tomato plants perceive reduced stress remains unclear (Szarka et al., 2012; El Arroussi et al., 2018). An almost similar trend in phytohormonal regulation was observed in salt-stressed wheat seedlings irrigated with *Chlorella* sp. HL water extract. In this case, jasmonic acid and cytokinin (zeatin) levels were reduced compared to untreated plants under salt stress, while salicylic acid, ABA, auxin (IAA), and gibberellic acid (GA_3_) levels were elevated (Liu et al., 2024). Salt stress typically decreases auxin and gibberellic acid levels, thereby inhibiting growth, development, and lateral root formation. Conversely, salicylic acid, which maintains glutathione levels and redox homeostasis, and ABA, which regulates stomatal closure and other abiotic stress resistance mechanisms, are often elevated under salt stress (Fu and Yang, 2023). The microalgal treatment in this study not only restored these hormone levels but, in some cases, enhanced them further, contributing to improved growth and stress tolerance in the wheat seedlings (Liu et al., 2024).

In conclusion, microalgal biostimulants demonstrate significant potential for mitigating salt stress in plants and ameliorating its adverse effects on general growth parameters (**Figure 1**). However, the precise mechanisms through which these biostimulants function remain elusive. Some studies suggest a role in alleviating osmotic stress, while others point to ionic stress mitigation, possibly through the activation of SOS pathways. This complexity extends to secondary stress responses such as oxidative stress. There is a pressing need for deeper investigation into these mechanisms, particularly through broader experimental parameters and advanced omics techniques. While lipid remodeling has been explored with promising insights, studies focusing on genetic, hormonal, and molecular mechanisms remain limited. Understanding how biostimulants interact with stress severity and plant species is key to optimizing their agricultural use.

## 5 Beat the heat: microalgae’s role in combatting heat stress in crops

Heat stress poses a critical challenge to global agriculture, with profound implications for food production and security. Anthropogenic activities have intensified greenhouse gas emissions, causing global temperatures to rise by 1.1°C above pre-industrial levels by 2021, with projections indicating an additional 1.5–2°C increase by the end of the century (Kumar and Kaushik, 2021). This warming trend severely threatens crop yields, with each 1°C rise reducing yields of wheat, rice, maize, and soybean by 6.0%, 3.2%, 7.4%, and 3.1%, respectively (Zhao et al., 2017). Moreover, increasing average temperatures and the rising frequency of heatwaves exacerbate water scarcity and drought stress, compounding agricultural challenges and exacerbating food insecurity. By 2050, climate-induced heat stress is projected to place an additional 8–80 million people at risk of hunger (Mbow et al., 2019).

Heat stress can disrupt plant morphology, physiology, and biochemistry, impacting both vegetative and generative stages. At the generative stage, elevated temperatures can impair pollen viability, reduce seed set and grain quality, and shorten ripening periods, ultimately diminishing yields (Fahad et al., 2017; Kumar and Kaushik, 2021). Photosynthesis is particularly vulnerable, as heat stress damages PSII, reduces ribulose-1,5-bisphosphate carboxylase/oxygenase activity, and impairs adenosine triphosphate synthesis, leading to decreased carbon fixation, energy production and ultimately biomass accumulation (Allakhverdiev et al., 2008). Additionally, increased transpiration under heat stress can accelerate water loss, disrupt membrane stability, and compromise metabolic function, further amplifying the physiological burden on plants (Hamilton et al., 2008). To counteract these effects, plants employ adaptive mechanisms such as heat shock proteins, which stabilize and refold denatured proteins, as well as antioxidant defenses, osmoprotectants, and hormonal responses involving ABA (Wang et al., 2004b; Wahid et al., 2007; Ahammed et al., 2016; Fahad et al., 2017). Despite the significant economic and societal implications of heat stress on crop production, the available literature on the effects of microalgal biostimulants in mitigating heat stress in plants is extremely limited. Notably, the only study identified on the subject did not explicitly aim to investigate heat stress. Kopta et al. (2018) evaluated the effects of a living *C. vulgaris* and bacteria consortium as a biostimulant on two lettuce cultivars (leafy and romaine) grown during the spring and summer seasons. Under optimal growth conditions during spring, the consortium increased yields by approximately 18.9%. During the summer, when average temperatures increased by 4°C, the yield of untreated lettuce declined by 10–30% compared to spring controls. In contrast, lettuce treated with the consortium maintained yields comparable to those achieved under spring conditions, despite the elevated temperatures. Additionally, in romaine lettuce grown during the summer, the biostimulant significantly enhanced total antioxidant activity and carotenoid levels compared to untreated controls. The scarcity of research on the role of microalgal biostimulants in addressing heat stress represents a significant gap in research. Given the critical nature of this abiotic stress and its projected impact on global food security, more focused studies are urgently needed to advance both scientific understanding and the practical use of microalgal biostimulants.

## 6 Bridging the gaps: advancing microalgal biostimulant research and application

The analysis of drought, salt, and heat stress highlights three critical gaps for the future research to address: the diversity gap, the practical gap, and the research gap.

Despite the vast diversity of microalgae, with an estimated 75,000 to 200,000 species in existence and approximately 45,000 cataloged (Guiry, 2012), *Arthrospira* sp. and *Chlorella* sp. dominate the literature as microalgal biostimulants for mitigating abiotic stress. This leaves a significant portion of microalgal diversity unexplored. To address this diversity gap, the development of high-throughput screening methods is crucial. Promising approaches include the yeast–Arabidopsis-based experiments of Saporta et al. (2019) and the Arabidopsis–lettuce-based methods of Chovanček et al. (2023), which have demonstrated their utility in identifying effective candidates. Beyond screening individual species, the strategic design of consortia composed of species with complementary modes of action could offer substantial benefits. For instance, Roque et al. (2023) evaluated a consortium of *Klebsormidium* sp., *Nostoc* sp., *Trichocoleus* sp., *Nodosilinea* sp., and *Microcoleus* sp. on *A. thaliana* and *Lolium multiflorum*. Their findings revealed notable improvements in growth, underscoring the potential of tailored microbial consortia in biostimulant applications.

In practice, drought, salt, and heat stress often occur simultaneously, creating complex challenges for crop productivity. This interconnected nature of abiotic stresses highlights the need for microalgal biostimulant studies that reflect real-world conditions. Research conducted on natural soils or under combinational stress conditions is essential to evaluate the actual effectiveness of these biostimulants. Although some studies, such as those investigating natural soils contaminated with salt and heavy metals, have begun to address this issue, much more work is needed to bridge this practical gap (Rady et al., 2023).

Another critical issue to address is the existing research gap. A noticeable trend in studies on drought and salt stress is their reliance on a narrow set of predefined biochemical parameters across different experiments. Commonly measured parameters include proline content, MDA, Na and K levels, and antioxidant activity, with limited exploration of additional indicators that could provide insights into the possible modes of action. To advance the field, there is an urgent need for comprehensive approaches, such as those offered by omics techniques. While these techniques have been widely adopted across various areas of scientific research, their application in the study of microalgal biostimulants remains limited (Mochida and Shinozaki, 2011; Maroli et al., 2018; Kimotho and Maina, 2024). Integrating omics approaches, including genomics, transcriptomics, proteomics, and metabolomics, can significantly enhance our understanding of the modes of action underlying biostimulants. Additionally, identifying the specific components in microalgae responsible for their biostimulant activity would be highly valuable. While substantial progress has been made in characterizing the chemical composition of microalgae and achieving their standardization, further exploration of specific fractions or compounds from microalgae is warranted. A deeper understanding of the molecular mechanisms in plants responding to microalgal treatments, as well as the specific compounds driving the biostimulant effect, could lead to improved efficacy, enhanced commercial viability, and increased trust among farmers in adopting these solutions.

## 7 Conclusion

Microalgal biostimulants are an interesting source for biostimulants due their sustainability, cultivation advantages and rich bioactive components. There is, however, still a lot to be left to research. Application methods, plant and algae species dependencies, and cultivation methods have a profound influence on abiotic stress mitigation. Despite these considerations, a plethora of studies have concluded that microalgal biostimulants can increase abiotic stress tolerance in a wide variety of plant species. Regarding drought stress, the effects on plants of microalgae are remarkably consistent across different species. They can mitigate yield losses, boost overall biomass accumulation, and restore nutrient uptake by enhancing photosynthetic efficiency and regulating stomatal function, which in turn improves water use efficiency. Biochemically, these biostimulants generally enhance drought tolerance through the accumulation of osmoprotectants and both enzymatic and non-enzymatic antioxidants. In contrast, salt stress mitigation using microalgal biostimulants seems highly dependent on the specific microalgal species. While all species exhibit some degree of morphological and physiological remediation in salt-stressed plants, their underlying biochemical responses differ. Some species enhance osmoprotectant and antioxidant accumulation, which is typically associated with improved stress tolerance, whereas others reduce these compounds. The reduction of these compounds has been linked to a decline in stress perception; however, the underlying biochemical mechanisms remain unclear. Heat stress, despite being a significant abiotic challenge, has been far less studied, representing still a considerable research gap. Overall, initial progress has already been made to increase our understanding about the effects of microalgae on plant growth and performance under different environmental conditions but further research is highly encouraged to unravel the different underlying modes of action.

## References

[B1] Ahammed G. J. Li X. Zhou J. Zhou Y. H. Yu J.-Q. (2016). “Role of hormones in plant adaptation to heat stress,” in Plant Hormones under Challenging Environmental Factors. Eds. Ahammed G. J. Yu J. Q. (Springer, Dordrecht), 1–23. doi: 10.1007/978-94-017-7758-2_1

[B2] Al Dayel M. F. El Sherif F. (2021). Evaluation of the effects of *Chlorella vulgaris*, *Nannochloropsis salina*, and *Enterobacter cloacae* on growth, yield and active compound compositions of *Moringa oleifera* under salinity stress. Saudi J. Biol. Sci. 28, 1687–1696. doi: 10.1016/j.sjbs.2020.12.007 33732054 PMC7938152

[B3] Allagulova C. R. Gimalov F. R. Shakirova F. M. Vakhitov V. A. (2003). The plant dehydrins: structure and putative functions. Biochem. (Mosc) 68, 945–951. doi: 10.1023/a:1026077825584 14606934

[B4] Allakhverdiev S. I. Kreslavski V. D. Klimov V. V. Los D. A. Carpentier R. Mohanty P. (2008). Heat stress: An overview of molecular responses in photosynthesis. Photosynth. Res. 98, 541–550. doi: 10.1007/s11120-008-9331-0 18649006

[B5] Alvarez A. L. Weyers S. L. Goemann H. M. Peyton B. M. Gardner R. D. (2021). Microalgae, soil and plants: A critical review of microalgae as renewable resources for agriculture. Algal. Res. 54, 102200. doi: 10.1016/j.algal.2021.102200

[B6] Arora N. K. (2019). Impact of climate change on agriculture production and its sustainable solutions. Environ. Sustain. 2, 95–96. doi: 10.1007/s42398-019-00078-w

[B7] Arya G. C. Sarkar S. Manasherova E. Aharoni A. Cohen H. (2021). The plant cuticle: an ancient guardian barrier set against long-standing rivals. Front. Plant Sci. 12. doi: 10.3389/fpls.2021.663165 PMC8267416 34249035

[B8] Bachani J. Mahanty A. Aftab T. Kumar K. (2022). Insight into calcium signalling in salt stress response. South Afr. J. Bot. 151, 1–8. doi: 10.1016/j.sajb.2022.09.033

[B9] Bahmani Jafarlou M. Pilehvar B. Modarresi M. Mohammadi M. (2021). Performance of algae extracts priming for enhancing seed germination indices and salt tolerance in *Calotropis procera* (Aiton) W.T. Iran J. Sci. Technol. Trans. A Sci. 45, 493–502. doi: 10.1007/s40995-021-01071-x

[B10] Barahoei M. Hatamipour M. S. Afsharzadeh S. (2021). Direct brackish water desalination using *Chlorella vulgaris* microalgae. Process Saf. Environ. Prot. 148, 237–248. doi: 10.1016/j.psep.2020.10.006

[B11] Barros-Rodríguez A. Rangseekaew P. Lasudee K. Pathom-Aree W. Manzanera M. (2021). Impacts of agriculture on the environment and soil microbial biodiversity. Plants 10 (11), 2325. doi: 10.3390/plants10112325 34834690 PMC8619008

[B12] Barsanti L. Coltelli P. Gualtieri P. (2019). Paramylon treatment improves quality profile and drought resistance in *Solanum lycopersicum* L. Cv. Micro-toM. Agronomy 9 (7), 394. doi: 10.3390/agronomy9070394

[B13] Bartley M. L. Boeing W. J. Corcoran A. A. Holguin F. O. Schaub T. (2013). Effects of salinity on growth and lipid accumulation of biofuel microalga *Nannochloropsis salina* and invading organisms. Biomass Bioenergy 54, 83–88. doi: 10.1016/j.biombioe.2013.03.026

[B14] Bayona-Morcillo P. J. Plaza B. M. Gómez-Serrano C. Rojas E. Jiménez-Becker S. (2020). Effect of the foliar application of cyanobacterial hydrolysate (Arthrospira platensis) on the growth of Petunia x hybrida under salinity conditions. J. Appl. Phycol. 32, 4003–4011. doi: 10.1007/s10811-020-02192-3/Published

[B15] Behera T. K. Krishna R. Ansari W. A. Aamir M. Kumar P. Kashyap S. P.. (2022). Approaches involved in the vegetable crops salt stress tolerance improvement: present status and way ahead. Front. Plant Sci. 12. doi: 10.3389/fpls.2021.787292 PMC8916085 35281697

[B16] Beillouin D. Ben-Ari T. Malézieux E. Seufert V. Makowski D. (2021). Positive but variable effects of crop diversification on biodiversity and ecosystem services. Glob. Chang. Biol. 27, 4697–4710. doi: 10.1111/gcb.15747 34114719

[B17] Bello A. S. Saadaoui I. Ben-Hamadou R. (2021). Beyond the source of bioenergy”: microalgae in modern agriculture as a biostimulant, biofertilizer, and anti-abiotic stress. Agronomy 11, 1610. doi: 10.3390/agronomy11081610

[B18] Berry Z. C. Emery N. C. Gotsch S. G. Goldsmith G. R. (2019). Foliar water uptake: Processes, pathways, and integration into plant water budgets. Plant Cell Environ. 42, 410–423. doi: 10.1111/pce.13439 30194766

[B19] Borowitzka M. A. (2018). “Biology of microalgae,” in Microalgae in Health and Disease Prevention. Eds. Levine I. A. Fleurence J. (Cambridge, Massachusetts (USA): Academic Press), 23–72. doi: 10.1016/B978-0-12-811405-6.00003-7

[B20] Carillo P. Ciarmiello L. F. Woodrow P. Corrado G. Chiaiese P. Rouphael Y. (2020). Enhancing sustainability by improving plant salt tolerance through macro-and micro-algal biostimulants. Biol. (Basel) 9, 1–21. doi: 10.3390/biology9090253 PMC7564450 32872247

[B21] Chew K. W. Chia S. R. Show P. L. Yap Y. J. Ling T. C. Chang J. S. (2018). Effects of water culture medium, cultivation systems and growth modes for microalgae cultivation: A review. J. Taiwan Inst. Chem. Eng. 91, 332–344. doi: 10.1016/j.jtice.2018.05.039

[B22] Chovanček E. Salazar J. Şirin S. Allahverdiyeva Y. (2023). Microalgae from Nordic collections demonstrate biostimulant effect by enhancing plant growth and photosynthetic performance. Physiol. Plant 175, e13911. doi: 10.1111/ppl.13911 37043258

[B23] Claeys H. Van Landeghem S. Dubois M. Maleux K. Inzé D. (2014). What Is Stress? Dose-response effects in commonly used *in vitro* stress assays. Plant Physiol. 165, 519–527. doi: 10.1104/pp.113.234641 24710067 PMC4044843

[B24] Clarke B. Otto F. Stuart-Smith R. Harrington L. (2022). Extreme weather impacts of climate change: an attribution perspective. Environ. Res.: Climate 1, 012001. doi: 10.1088/2752-5295/ac6e7d

[B25] Colla G. Rouphael Y. (2020). Microalgae: new source of plant biostimulants. Agronomy 10 (9), 1240. doi: 10.3390/agronomy10091240

[B26] Crist E. Mora C. Engelman R. (2017). The interaction of human population, food production, and biodiversity protection. Sci. (1979) 356, 260–264. doi: 10.1126/science.aal2011 28428391

[B27] De Azevedo Neto A. D. Prisco J. T. Enéas-Filho J. De Abreu C. E. B. Gomes-Filho E. (2006). Effect of salt stress on antioxidative enzymes and lipid peroxidation in leaves and roots of salt-tolerant and salt-sensitive maize genotypes. Environ. Exp. Bot. 56, 87–94. doi: 10.1016/j.envexpbot.2005.01.008

[B28] Deeba F. Pandey A. K. Ranjan S. Mishra A. Singh R. Sharma Y. K.. (2012). Physiological and proteomic responses of cotton (Gossypium herbaceum L.) to drought stress. Plant Physiol. Biochem. 53, 6–18. doi: 10.1016/j.plaphy.2012.01.002 22285410

[B29] De La Reguera E. Veatch J. Gedan K. Tully K. L. (2020). The effects of saltwater intrusion on germination success of standard and alternative crops. Environ. Exp. Bot. 180. doi: 10.1016/j.envexpbot.2020.104254

[B30] Devika O. S. Singh S. Sarkar D. Barnwal P. Suman J. Rakshit A. (2021). Seed priming: A potential supplement in integrated resource management under fragile intensive ecosystems. Front. Sustain Food Syst. 5. doi: 10.3389/fsufs.2021.654001

[B31] Dinh T. H. Takaragawa H. Watanabe K. Nakabaru M. Kawamitsu Y. (2019). Leaf photosynthesis response to change of soil moisture content in sugarcane. Sugar Tech. 21, 949–958. doi: 10.1007/s12355-019-00735-8

[B32] du Jardin P. (2015). Plant biostimulants: Definition, concept, main categories and regulation. Sci. Hortic. 196, 3–14. doi: 10.1016/j.scienta.2015.09.021

[B33] El Arroussi H. Benhima R. Elbaouchi A. Sijilmassi B. EL Mernissi N. Aafsar A.. (2018). Dunaliella salina exopolysaccharides: a promising biostimulant for salt stress tolerance in tomato (*Solanum lycopersicum*). J. Appl. Phycol. 30, 2929–2941. doi: 10.1007/s10811-017-1382-1

[B34] El Arroussi H. Elbaouchi A. Benhima R. Bendaou N. Smouni A. Wahby I. (2016). “Halophilic microalgae Dunaliella salina extracts improve seed germination and seedling growth of Triticum aestivum L. under salt stress,” in Acta Horticulturae (: Leuven, Belgium International Society for Horticultural Science), 13–26. doi: 10.17660/ActaHortic.2016.1148.2

[B35] El-Baky H. H. A. El-Baz F. K. Baroty G. S. E. (2010). Enhancing antioxidant availability in wheat grains from plants grown under seawater stress in response to microalgae extract treatments. J. Sci. Food Agric. 90, 299–303. doi: 10.1002/jsfa.3815 20355046

[B36] Elmenofy H. M. Hatterman-Valenti H. M. Hassan I. F. Mahmoud M. M. A. (2023). Effects of deficit irrigation and anti-stressors on water productivity, and fruit quality at harvest and stored ‘Murcott’ Mandarin. Horticulturae 9 (7), 787. doi: 10.3390/horticulturae9070787

[B37] Elnajar M. Aldesuquy H. Abdelmoteleb M. Eltanahy E. (2024). Mitigating drought stress in wheat plants (Triticum Aestivum L.) through grain priming in aqueous extract of spirulina platensis. BMC Plant Biol. 24, 233. doi: 10.1186/s12870-024-04905-z 38561647 PMC10986097

[B38] Escalante F. M. E. Cortés-Jiménez D. Tapia-Reyes G. Suárez R. (2015). Immobilized microalgae and bacteria improve salt tolerance of tomato seedlings grown hydroponically. J. Appl. Phycol. 27, 1923–1933. doi: 10.1007/s10811-015-0651-0

[B39] Fahad S. Bajwa A. A. Nazir U. Anjum S. A. Farooq A. Zohaib A.. (2017). Crop production under drought and heat stress: Plant responses and management options. Front. Plant Sci. 8. doi: 10.3389/fpls.2017.01147 PMC5489704 28706531

[B40] FAO (2022). Global Symposium on Salt-Affected Soils: Outcome document (Rome: FAO). doi: 10.4060/cb9929en

[B41] FAO IFAD UNICEF WFP WHO (2023). The State of Food Security and Nutrition in the World 2023: Urbanization, agrifood systems transformation and healthy diets across the rural–urban continuum (Rome: FAO; IFAD; UNICEF; WFP; WHO). doi: 10.4060/cc3017en

[B42] Farhat N. Rabhi M. Falleh H. Jouini J. Abdelly C. Smaoui A. (2011). Optimization of salt concentrations for a higher carotenoid production in dunaliella salina (Chlorophyceae). J. Phycol. 47, 1072–1077. doi: 10.1111/j.1529-8817.2011.01036.x 27020189

[B43] Farooq M. Wahid A. Kobayashi N. Fujita D. Basra S. M. A. (2009). Plant drought stress: Effects, mechanisms and management. Agron. Sustain Dev. 29, 185–212. doi: 10.1051/agro:2008021

[B44] Fu H. Yang Y. (2023). How plants tolerate salt stress. Curr. Issues Mol. Biol. 45, 5914–5934. doi: 10.3390/cimb45070374 37504290 PMC10378706

[B45] Galli V. da Silva Messias R. Perin E. C. Borowski J. M. Bamberg A. L. Rombaldi C. V. (2016). Mild salt stress improves strawberry fruit quality. LWT 73, 693–699. doi: 10.1016/j.lwt.2016.07.001

[B46] Gitau M. M. Shetty P. Maróti G. (2023). Transcriptional analysis reveals induction of systemic resistance in tomato treated with Chlorella microalgae. Algal. Res. 72, 103106. doi: 10.1016/j.algal.2023.103106

[B47] Gogna M. Choudhary A. Mishra G. Kapoor R. Bhatla S. C. (2020). Changes in lipid composition in response to salt stress and its possible interaction with intracellular Na+-K+ ratio in sunflower (Helianthus annuus L.). Environ. Exp. Bot. 178, 104147. doi: 10.1016/j.envexpbot.2020.104147

[B48] Gonzales Cruz C. Centeno da Rosa A. P. Strentzle B. R. Vieira Costa J. A. (2023). Microalgae-based dairy effluent treatment coupled with the production of agricultural biostimulant. J. Appl. Phycol. 35, 2881–2890. doi: 10.1007/s10811-023-03091-z

[B49] Guiry M. D. (2012). How many species of algae are there? J. Phycol. 48, 1057–1063. doi: 10.1111/j.1529-8817.2012.01222.x 27011267

[B50] Gurrieri L. Merico M. Trost P. Forlani G. Sparla F. (2020). Impact of drought on soluble sugars and free proline content in selected arabidopsis mutants. Biol. (Basel) 9, 1–14. doi: 10.3390/biology9110367 PMC7692697 33137965

[B51] Guzmán-Murillo M. A. Ascencio F. Larrinaga-Mayoral J. A. (2013). Germination and ROS detoxification in bell pepper (Capsicum annuum L.) under NaCl stress and treatment with microalgae extracts. Protoplasma 250, 33–42. doi: 10.1007/s00709-011-0369-z 22234834

[B52] Hamilton E. W. Heckathorn S. A. Joshi P. Wang D. Barua D. (2008). Interactive effects of elevated CO2 and growth temperature on the tolerance of photosynthesis to acute heat stress in C3 and C4 species. J. Integr. Plant Biol. 50, 1375–1387. doi: 10.1111/j.1744-7909.2008.00747.x 19017125

[B53] Hamouda R. A. Shehawy M. A. Mohy El Din S. M. Albalwe F. M. Albalawi H. M. R. Hussein M. H. (2022). Protective role of Spirulina platensis liquid extract against salinity stress effects on Triticum aestivum L. Green Process. Synthesis 11, 648–658. doi: 10.1515/gps-2022-0065

[B54] Hara M. Fujinaga M. Kuboi T. (2004). Radical scavenging activity and oxidative modification of citrus dehydrin. Plant Physiol. Biochem. 42, 657–662. doi: 10.1016/j.plaphy.2004.06.004 15331095

[B55] Ibañez E. Herrero M. Mendiola J. A. Castro-Puyana M. (2012). “Extraction and characterization of bioactive compounds with health benefits from marine resources: macro and micro algae, cyanobacteria, and invertebrates,” in Marine Bioactive Compounds. Ed. Hayes M. (Springer, Berlin/Heidelberg, Germany), 55–98. doi: 10.1007/978-1-4614-1247-2_2

[B56] Ishfaq M. Kiran A. ur Rehman H. Farooq M. Ijaz N. H. Nadeem F.. (2022). Foliar nutrition: Potential and challenges under multifaceted agriculture. Environ. Exp. Bot. 200, 104909. doi: 10.1016/j.envexpbot.2022.104909

[B57] Kanchan A. Simranjit K. Ranjan K. Prasanna R. Ramakrishnan B. Singh M. C.. (2019). Microbial biofilm inoculants benefit growth and yield of chrysanthemum varieties under protected cultivation through enhanced nutrient availability. Plant Biosyst. 153, 306–316. doi: 10.1080/11263504.2018.1478904

[B58] Kapazoglou A. Gerakari M. Lazaridi E. Kleftogianni K. Sarri E. Tani E.. (2023). Crop wild relatives: A valuable source of tolerance to various abiotic stresses. Plants 12 (2), 328. doi: 10.3390/plants12020328 36679041 PMC9861506

[B59] Kapoore R. V. Wood E. E. Llewellyn C. A. (2021). Algae biostimulants: A critical look at microalgal biostimulants for sustainable agricultural practices. Biotechnol. Adv. 49, 107754. doi: 10.1016/j.bioteChadv.2021.107754 33892124

[B60] Keo V. Kaosol T. (2020). Effect of salinity on *Chlorella vulgaris* for increasing lipid content. Thai Environ. Eng. J. 34, 11–21.

[B61] Kim W. Iizumi T. Nishimori M. (2019). Global patterns of crop production losses associated with droughts from 1983 to 2009. J. Appl. Meteorol. Climatol. 58, 1233–1244. doi: 10.1175/JAMC-D-18-0174.1

[B62] Kimotho R. N. Maina S. (2024). Unraveling plant–microbe interactions: can integrated omics approaches offer concrete answers? J. Exp. Bot. 75, 1289–1313. doi: 10.1093/jxb/erad448 37950741 PMC10901211

[B63] Kopta T. Pavlíková M. Skara A. Pokluda R. Maršálek B. (2018). Effect of bacterial-algal biostimulant on the yield and internal quality of Lettuce (Lactuca sativa L.) produced for spring and summer crop. Not Bot. Horti. Agrobot. Cluj. Napoca 46, 615–621. doi: 10.15835/nbha46211110

[B64] Kumar A. Kaushik P. (2021). Heat Stress and its Impact on Plant Function: An Update. (Basel, Switzerland: MDPI (Multidisciplinary Digital Publishing Institute)). doi: 10.20944/preprints202108.0489.v1

[B65] Kumar S. Wani A. W. Kaushik R. Kaur H. Djajadi D. Khamidah A.. (2024). Navigating the landscape of precision horticulture: Sustainable agriculture in the digital Age. Sci. Hortic. 338, 113688. doi: 10.1016/j.scienta.2024.113688

[B66] Kusvuran S. (2021). Microalgae (*Chlorella vulgaris* Beijerinck) alleviates drought stress of broccoli plants by improving nutrient uptake, secondary metabolites, and antioxidative defense system. Hortic. Plant J. 7, 221–231. doi: 10.1016/j.hpj.2021.03.007

[B67] Kusvuran A. Can A. G. (2020). Effects of microalga (*Chlorella vulgaris* beijerinck) on seconder metabolites and antioxidative defense system improve plant growth and salt tolerance in guar [Cyamopsis tetragonoloba (L.) taub. Legume Res. 43, 56–60. doi: 10.18805/LR-492

[B68] Kusvuran A. Kusvuran S. (2019). Using of microbial fertilizer as biostimulant alleviates damage from drought stress in guar (*Cyamopsis tetragonoloba* (L.) taub.) seedlings. Int. Lett. Natural Sci. 76, 147–157. doi: 10.56431/p-x0z5sx

[B69] Lal R. (2021). Feeding the world and returning half of the agricultural land back to nature. J. Soil Water Conserv. 76, 75–78. doi: 10.2489/JSWC.2021.0607A

[B70] Li Y. Xu S. S. Gao J. Pan S. Wang G. X. (2014). Chlorella induces stomatal closure via NADPH oxidase-dependent ROS production and its effects on instantaneous water use efficiency in Vicia faba. PloS One 9 (3), e93290. doi: 10.1371/journal.pone.0093290 24687099 PMC3970962

[B71] Liu X. Y. Hong Y. Zhang Y. W. Li L. H. (2024). Valorization of treated swine wastewater and generated biomass by microalgae: Their effects and salt tolerance mechanisms on wheat seedling growth. Environ. Res. 251, 118664. doi: 10.1016/j.envres.2024.118664 38499222

[B72] Ljubic A. Holdt S. L. Jakobsen J. Bysted A. Jacobsen C. (2021). Fatty acids, carotenoids, and tocopherols from microalgae: targeting the accumulation by manipulating the light during growth. J. Appl. Phycol. 33, 2783–2793. doi: 10.1007/s10811-021-02503-2

[B73] Ma C. Cui H. Ren C. Yang J. Liu Z. Tang T.. (2022). The seed primer and biofertilizer performances of living *Chlorella pyrenoidosa* on Chenopodium quinoa under saline-alkali condition. J. Appl. Phycol. 34, 1621–1634. doi: 10.1007/s10811-022-02699-x

[B74] Ma X. Zheng J. Zhang X. Hu Q. Qian R. (2017). Salicylic acid alleviates the adverse effects of salt stress on dianthus superbus (Caryophyllaceae) by activating photosynthesis, protecting morphological structure, and enhancing the antioxidant system. Front. Plant Sci. 8. doi: 10.3389/fpls.2017.00600 PMC5399920 28484476

[B75] Maroli A. S. Gaines T. A. Foley M. E. Duke S. O. Doǧramacl M. Anderson J. V.. (2018). Omics in weed science: A perspective from genomics, transcriptomics, and metabolomics approaches. Weed Sci. 66, 681–695. doi: 10.1017/wsc.2018.33

[B76] Marques H. M. C. Mógor Á. F. Amatussi J. O. de Lara G. B. Mógor G. Sant’Anna-Santos B. F. (2023). Use of microalga Asterarcys quadricellularis in common bean. J. Appl. Phycol. 35, 2891–2905. doi: 10.1007/s10811-023-03098-6

[B77] Martini F. Beghini G. Zanin L. Varanini Z. Zamboni A. Ballottari M. (2021). The potential use of Chlamydomonas reinhardtii and *Chlorella sorokiniana* as biostimulants on maize plants. Algal. Res. 60, 102515. doi: 10.1016/j.algal.2021.102515 34745855 PMC7611950

[B78] Martin-StPaul N. Delzon S. Cochard H. (2017). Plant resistance to drought depends on timely stomatal closure. Ecol. Lett. 20, 1437–1447. doi: 10.1111/ele.12851 28922708

[B79] Mbow C. Rosenzweig C. Barioni L. G. Benton T. G. Herrero M. Krishnapillai M.. (2019). “Food security,” in Climate Change and Land: An IPCC Special Report on climate change, desertification, land degradation, sustainable land management, food security, and greenhouse gas fluxes in terrestrial ecosystems. Eds. Shukla P. R. Skea J. Buendia E.C. Masson-Delmotte V. Pörtner H.-O. Roberts D. C. (Cambridge, United Kingdom: Cambridge University Press), 437–550. doi: 10.1017/9781009157988.007

[B80] Michalak I. Chojnacka K. (2014). Algal extracts: Technology and advances. Eng. Life Sci. 14, 581–591. doi: 10.1002/elsc.201400139

[B81] Mignolet-Spruyt L. Xu E. Idänheimo N. Hoeberichts F. A. Mühlenbock P. Brosche M.. (2016). Spreading the news: Subcellular and organellar reactive oxygen species production and signalling. J. Exp. Bot. 67, 3831–3844. doi: 10.1093/jxb/erw080 26976816

[B82] Moayedi A. Yargholi B. Pazira E. Babazadeh H. (2019). Investigated of desalination of saline waters by using dunaliella salina algae and its effect on water ions. Civil Eng. J. (Iran) 5, 2450–2460. doi: 10.28991/cej-2019-03091423

[B83] Mochida K. Shinozaki K. (2011). Advances in omics and bioinformatics tools for systems analyses of plant functions. Plant Cell Physiol. 52, 2017–2038. doi: 10.1093/pcp/pcr153 22156726 PMC3233218

[B84] Molino A. Mehariya S. Iovine A. Casella P. Marino T. Karatza D.. (2020). Enhancing biomass and lutein production from scenedesmus almeriensis: effect of carbon dioxide concentration and culture medium reuse. Front. Plant Sci. 11. doi: 10.3389/fpls.2020.00415 PMC7186383 32373140

[B85] Moon J. Park Y. J. Choi Y. Truong T. Q. Huynh P. K. Kim Y. B.. (2024). Physiological effects and mechanisms of *Chlorella vulgaris* as a biostimulant on the growth and drought tolerance of *Arabidopsis thaliana* . Plants 13 (21), 3012. doi: 10.3390/plants13213012 39519931 PMC11548328

[B86] Mostafa M. M. Hammad D. M. Reda M. M. El-Sayed A. E. K. B. (2023). Water extracts of Spirulina platensis and *Chlorella vulgaris* enhance tomato (*Solanum lycopersicum* L.) tolerance against saline water irrigation. Biomass Convers. Biorefin. 14, 21181–21191. doi: 10.1007/s13399-023-04460-x

[B87] Munns R. (2002). Comparative physiology of salt and water stress. Plant Cell Environ. 25, 239–250. doi: 10.1046/j.0016-8025.2001.00808.x 11841667

[B88] Mutale-joan C. Rachidi F. Mohamed H. A. Mernissi N. Aasfar A. Barakate M.. (2021). Microalgae-cyanobacteria–based biostimulant effect on salinity tolerance mechanisms, nutrient uptake, and tomato plant growth under salt stress. J. Appl. Phycol. 33, 3779–3795. doi: 10.1007/s10811-021-02559-0

[B89] Mutale-joan C. Redouane B. Najib E. Yassine K. Lyamlouli K. Laila S.. (2020). Screening of microalgae liquid extracts for their bio stimulant properties on plant growth, nutrient uptake and metabolite profile of *Solanum lycopersicum* L. Sci. Rep. 10, 2820. doi: 10.1038/s41598-020-59840-4 32071360 PMC7028939

[B90] Mutale-Joan C. Sbabou L. Hicham E. A. (2023). Microalgae and cyanobacteria: how exploiting these microbial resources can address the underlying challenges related to food sources and sustainable agriculture: A review. J. Plant Growth Regul. 42, 1–20. doi: 10.1007/s00344-021-10534-9

[B91] Nadarajah K. K. (2020). ROS homeostasis in abiotic stress tolerance in plants. Int. J. Mol. Sci. 21, 1–29. doi: 10.3390/ijms21155208 PMC7432042 32717820

[B92] Navarro-López E. Ruiz-Nieto A. Gallardo-Rodríguez J. J. Cerón-García M. C. González-López C. V. Acién-Fernández F. G. (2023). Downstream processing of Scenedesmus sp. to obtain biostimulants. J. Appl. Phycol. 35, 2193–2203. doi: 10.1007/s10811-023-03039-3

[B93] Negrão S. Schmöckel S. M. Tester M. (2017). Evaluating physiological responses of plants to salinity stress. Ann. Bot. 119, 1–11. doi: 10.1093/aob/mcw191 27707746 PMC5218372

[B94] Netting A. G. (2000). pH, abscisic acid and the integration of metabolism in plants under stressed and non-stressed conditions: cellular responses to stress and their implication for plant water relations. J. Exp. Bot. 51, 147–158. doi: 10.1093/jexbot/51.343.147 10938821

[B95] Oancea F. Velea S. Fatu V. Mincea C. Ilie L. (2013). Micro-algae based plant biostimulant and its effect on water stressed tomato plants. Romanian J. Plant Prot. 6, 104–117.

[B96] Oral E. Tunçtürk R. Tunçtürk M. (2021). The Effect of Rhizobacteria in the Reducing drought Stress in Soybean (Glycine max L.). Legume Res. 44, 1172–1178. doi: 10.18805/LR-631

[B97] Özdemir S. Sukatar A. Öztekin G. B. (2016). Production of *Chlorella vulgaris* and its effects on plant growth, yield and fruit quality of organic tomato grown in greenhouse as biofertilizer. Tarim Bilimleri Dergisi 22, 596–605. doi: 10.1501/tarimbil_0000001418

[B98] Panta S. Flowers T. Lane P. Doyle R. Haros G. Shabala S. (2014). Halophyte agriculture: Success stories. Environ. Exp. Bot. 107, 71–83. doi: 10.1016/j.envexpbot.2014.05.006

[B99] Parmar P. Kumar R. Neha Y. Srivatsan V. (2023). Microalgae as next generation plant growth additives: Functions, applications, challenges and circular bioeconomy based solutions. Front. Plant Sci. 14. doi: 10.3389/fpls.2023.1073546 PMC10101342 37063190

[B100] Pinke Z. Ács T. Kalicz P. Kern Z. Jambor A. (2024). Hotspots in the EU-27 and economic consequences of the 2022 spring-summer drought. EuroChoices 23, 28–33. doi: 10.1111/1746-692X.12423

[B101] Plaza B. M. Gómez-Serrano C. Acién-Fernández F. G. Jimenez-Becker S. (2018). Effect of microalgae hydrolysate foliar application (Arthrospira platensis and Scenedesmus sp.) on Petunia x hybrida growth. J. Appl. Phycol. 30, 2359–2365. doi: 10.1007/s10811-018-1427-0

[B102] Pokhrel Y. Felfelani F. Satoh Y. Boulange J. Burek P. Gädeke A.. (2021). Global terrestrial water storage and drought severity under climate change. Nat. Clim. Chang. 11, 226–233. doi: 10.1038/s41558-020-00972-w

[B103] Prăvălie R. Patriche C. Borrelli P. Panagos P. Roşca B. Dumitraşcu M.. (2021). Arable lands under the pressure of multiple land degradation processes. A global perspective. Environ. Res. 194, 110697. doi: 10.1016/j.envres.2020.110697 33428912

[B104] Prisa D. Spagnuolo D. (2023). Plant production with microalgal biostimulants. Horticulturae 9 (7), 829. doi: 10.3390/horticulturae9070829

[B105] Puglisi I. La Bella E. Rovetto E. I. Stevanato P. Fascella G. Baglieri A. (2022). Morpho-biometric and biochemical responses in lettuce seedlings treated by different application methods of *Chlorella vulgaris* extract: foliar spray or root drench? J. Appl. Phycol. 34, 889–901. doi: 10.1007/s10811-021-02671-1

[B106] Qu D. Show P. L. Miao X. (2021). Transcription factor chbzip1 from alkaliphilic microalgae Chlorella sp. Bld enhancing alkaline tolerance in transgenic arabidopsis thaliana. Int. J. Mol. Sci. 22, 1–16. doi: 10.3390/ijms22052387 PMC7957498 33673599

[B107] Rady M. M. Elrys A. S. Selem E. Mohsen A. A. A. Arnaout S. M. A. I. El-Sappah A. H.. (2023). Spirulina platensis extract improves the production and defenses of the common bean grown in a heavy metals-contaminated saline soil. J. Environ. Sci. (China) 129, 240–257. doi: 10.1016/j.jes.2022.09.011 36804239

[B108] Ramluckan K. Moodley K. G. Bux F. (2014). An evaluation of the efficacy of using selected solvents for the extraction of lipids from algal biomass by the soxhlet extraction method. Fuel 116, 103–108. doi: 10.1016/j.fuel.2013.07.118

[B109] Ranglová K. Lakatos G. E. Câmara Manoel J. A. Grivalský T. Suárez Estrella F. Acién Fernández F. G.. (2021). Growth, biostimulant and biopesticide activity of the MACC-1 Chlorella strain cultivated outdoors in inorganic medium and wastewater. Algal. Res. 53, 102136. doi: 10.1016/j.algal.2020.102136

[B110] Reganold J. P. Wachter J. M. (2016). Organic agriculture in the twenty-first century. Nat. Plants 2, 15221. doi: 10.1038/nplants.2015.221 27249193

[B111] Renuka N. Guldhe A. Prasanna R. Singh P. Bux F. (2018). Microalgae as multi-functional options in modern agriculture: current trends, prospects and challenges. Biotechnol. Adv. 36, 1255–1273. doi: 10.1016/j.bioteChadv.2018.04.004 29673972

[B112] Ritchie H. Roser M. (2019). Half of the world’s habitable land is used for agriculture (OurWorldinData.org). Available online at: https://ourworldindata.org/global-land-for-agriculture (Accessed January 24, 2025).

[B113] Rocha I. Ma Y. Souza-Alonso P. Vosátka M. Freitas H. Oliveira R. S. (2019). Seed coating: A tool for delivering beneficial microbes to agricultural crops. Front. Plant Sci. 10. doi: 10.3389/fpls.2019.01357 PMC6852281 31781135

[B114] Rong L.b. Gong K.y. Duan F.y. Li S.k. Zhao M. He J.. (2021). Yield gap and resource utilization efficiency of three major food crops in the world – A review. J. Integr. Agric. 20, 349–362. doi: 10.1016/S2095-3119(20)63555-9

[B115] Roque J. Brito Â. Rocha M. Pissarra J. Nunes T. Bessa M.. (2023). Isolation and characterization of soil cyanobacteria and microalgae and evaluation of their potential as plant biostimulants. Plant Soil 493, 115–136. doi: 10.1007/s11104-023-06217-x

[B116] Ruiz-Domínguez M. C. Medina E. Salinas F. Bugueño W. Fuentes J. L. Vílchez C.. (2022). Methodological optimization of supercritical fluid extraction of valuable bioactive compounds from the acidophilic microalga coccomyxa onubensis. Antioxidants 11 (7), 1248. doi: 10.3390/antiox11071248 35883739 PMC9312109

[B117] Safi C. Zebib B. Merah O. Pontalier P. Y. Vaca-Garcia C. (2014). Morphology, composition, production, processing and applications of *Chlorella vulgaris*: A review. Renewable Sustain. Energy Rev. 35, 265–278. doi: 10.1016/j.rser.2014.04.007

[B118] Salih S. Bahjat N. Tuncturk R. (2022). Enhancing *in vitro* Growth of Wheat Seedlings Under Water Stress using Biopriming. J. Agric. Sci. - Sri Lanka 17, 437–444. doi: 10.4038/jas.v17i3.9923

[B119] Salvi L. Niccolai A. Cataldo E. Sbraci S. Paoli F. Storchi P.. (2020). Effects of Arthrospira platensis Extract on Physiology and Berry Traits in Vitis vinifera. Plants 9, 1–13. doi: 10.3390/plants9121805 PMC7766242 33352675

[B120] Sánchez-Quintero Á. Fernandes S. C. M. Beigbeder J. B. (2023). Overview of microalgae and cyanobacteria-based biostimulants produced from wastewater and CO2 streams towards sustainable agriculture: A review. Microbiol. Res. 277, 127505. doi: 10.1016/j.micres.2023.127505 37832502

[B121] Santoro D. F. Puglisi I. Sicilia A. Baglieri A. La Bella E. Lo Piero A. R. (2023). Transcriptomic profile of lettuce seedlings (Lactuca sativa) response to microalgae extracts used as biostimulant agents. AoB Plants 15 (4), plad043. doi: 10.1093/aobpla/plad043 37434759 PMC10332502

[B122] Saporta R. Bou C. Frías V. Mulet J. M. (2019). A method for a fast evaluation of the biostimulant potential of different natural extracts for promoting growth or tolerance against abiotic stress. Agronomy 9 (3), 143. doi: 10.3390/agronomy9030143

[B123] Selem E. E. S. (2019). Physiological effects of spirulina platensis in salt stressed vicia faba l. Plants. Egyptian J. Bot. 59, 185–194. doi: 10.21608/ejbo.2018.3836.1178

[B124] Selem E. Tuncturk R. Nohutcu L. Tuncturk M. (2022). Effects of rhizobacteria and algal species on physiological and biochemical parameters in Calendula officinalis L. under different irrigation regimes. J. Elem. 27, 87–97. doi: 10.5601/jelem.2022.27.1.2173

[B125] Sharma H. S. S. Fleming C. Selby C. Rao J. R. Martin T. (2014). Plant biostimulants: A review on the processing of macroalgae and use of extracts for crop management to reduce abiotic and biotic stresses. J. Appl. Phycol. 26, 465–490. doi: 10.1007/s10811-013-0101-9

[B126] Silva E. N. Ferreira-Silva S. L. Viégas R. A. Silveira J. A. G. (2010). The role of organic and inorganic solutes in the osmotic adjustment of drought-stressed Jatropha curcas plants. Environ. Exp. Bot. 69, 279–285. doi: 10.1016/j.envexpbot.2010.05.001

[B127] Soulages J. L. Kim K. Arrese E. L. Walters C. Cushman J. C. (2003). Conformation of a group 2 late embryogenesis abundant protein from soybean. Evidence of poly (L-proline)-type II structure. Plant Physiol. 131, 963–975. doi: 10.1104/pp.015891 12644649 PMC166862

[B128] Stirk W. A. Bálint P. Vambe M. Lovász C. Molnár Z. van Staden J.. (2020). Effect of cell disruption methods on the extraction of bioactive metabolites from microalgal biomass. J. Biotechnol. 307, 35–43. doi: 10.1016/j.jbiotec.2019.10.012 31678206

[B129] Su M. Bastiaens L. Verspreet J. Hayes M. (2023). Applications of microalgae in foods, pharma and feeds and their use as fertilizers and biostimulants: legislation and regulatory aspects for consideration. Foods 12 (20), 3878. doi: 10.3390/foods12203878 37893770 PMC10606004

[B130] Suchithra M. R. Muniswami D. M. Sri M. S. Usha R. Rasheeq A. A. Preethi B. A.. (2022). Effectiveness of green microalgae as biostimulants and biofertilizer through foliar spray and soil drench method for tomato cultivation. South Afr. J. Bot. 146, 740–750. doi: 10.1016/j.sajb.2021.12.022

[B131] Szarka A. Tomasskovics B. Bánhegyi G. (2012). The ascorbate-glutathione-α-tocopherol triad in abiotic stress response. Int. J. Mol. Sci. 13, 4458–4483. doi: 10.3390/ijms13044458 22605990 PMC3344226

[B132] Tejada-Ruiz S. Gonzalez-Lopez C. Rojas E. Jiménez-Becker S. (2020). Effect of the foliar application of microalgae hydrolysate (Arthrospira platensis) and silicon on the growth of pelargonium hortorum L.H. Bailey under salinity conditions. Agronomy 10 (11), 1713. doi: 10.3390/agronomy10111713

[B133] Trejo A. de-Bashan L. E. Hartmann A. Hernandez J. P. Rothballer M. Schmid M.. (2012). Recycling waste debris of immobilized microalgae and plant growth-promoting bacteria from wastewater treatment as a resource to improve fertility of eroded desert soil. Environ. Exp. Bot. 75, 65–73. doi: 10.1016/j.envexpbot.2011.08.007

[B134] van Dijk M. Morley T. Rau M. L. Saghai Y. (2021). A meta-analysis of projected global food demand and population at risk of hunger for the period 2010–2050. Nat. Food 2, 494–501. doi: 10.1038/s43016-021-00322-9 37117684

[B135] Vuppaladadiyam A. K. Prinsen P. Raheem A. Luque R. Zhao M. (2018). Microalgae cultivation and metabolites production: a comprehensive review. Biofuels Bioprod. Biorefining 12, 304–324. doi: 10.1002/bbb.1864

[B136] Wahab A. Abdi G. Saleem M. H. Ali B. Ullah S. Shah W.. (2022). Plants’ Physio-biochemical and phyto-hormonal responses to alleviate the adverse effects of drought stress: A comprehensive review. Plants 11 (13), 1620. doi: 10.3390/plants11131620 35807572 PMC9269229

[B137] Wahid A. Gelani S. Ashraf M. Foolad M. R. (2007). Heat tolerance in plants: An overview. Environ. Exp. Bot. 61, 199–223. doi: 10.1016/j.envexpbot.2007.05.011

[B138] Wang H. Li C. Pu J. Zhou S. Wu Y. Guo F.. (2024). The culture of Chlorella with livestock wastewater and the application of its oligosaccharides in promoting rice seed germination under high salinity conditions. Biochem. Eng. J. 209, 109399. doi: 10.1016/j.bej.2024.109399

[B139] Wang C. Linderholm H. W. Song Y. Wang F. Liu Y. Tian J.. (2020). Impacts of drought on maize and soybean production in northeast China during the past five decades. Int. J. Environ. Res. Public Health 17 (7), 2459. doi: 10.3390/ijerph17072459 32260284 PMC7177764

[B140] Wang W. Vinocur B. Shoseyov O. Altman A. (2004b). Role of plant heat-shock proteins and molecular chaperones in the abiotic stress response. Trends Plant Sci. 9, 244–252. doi: 10.1016/j.tplants.2004.03.006 15130550

[B141] Wang S. Wan C. Wang Y. Chen H. Zhou Z. Fu H.. (2004a). The characteristics of Na+, K+ and free proline distribution in several drought-resistant plants of the Alxa Desert, China. J. Arid. Environ. 56, 525–539. doi: 10.1016/S0140-1963(03)00063-6

[B142] Wei J. Gao L. Shen G. Yang X. Li M. (2020). The role of adsorption in microalgae biological desalination: Salt removal from brackish water using Scenedesmus obliquus. Desalination 493, 114616. doi: 10.1016/j.desal.2020.114616

[B143] Werner C. Correia O. Beyschlag W. (1999). Two different strategies of Mediterranean macchia plants to avoid photoinhibitory damage by excessive radiation levels during summer drought. Acta Oecologica 20, 15–23. doi: 10.1016/S1146-609X(99)80011-3

[B144] Wing I. S. De Cian E. Mistry M. N. (2021). Global vulnerability of crop yields to climate change. J. Environ. Econ. Manage 109, 102462. doi: 10.1016/j.jeem.2021.102462

[B145] Yang Y. Guo Y. (2018). Unraveling salt stress signaling in plants. J. Integr. Plant Biol. 60, 796–804. doi: 10.1111/jipb.12689 29905393

[B146] Yang X. Lu M. Wang Y. Wang Y. Liu Z. Chen S. (2021). Response mechanism of plants to drought stress. Horticulturae 7 (3), 50. doi: 10.3390/horticulturae7030050

[B147] Yolci M. S. Tunçtürk R. Tunçtürk M. Ceylan S. Arvas Y. E. (2022). Effect of Rhizobacteria and Microalgae Treatments on Some Physiological and Biochemical Parameters of Fenugreek (Trigonella foenum-graecum L.) Grown under Drought Stress. Legume Res. 45, 415–421. doi: 10.18805/LRF-675

[B148] Yuan X. Li Y. Liu S. Xia F. Li X. Qi B. (2014). Accumulation of eicosapolyenoic acids enhances sensitivity to abscisic acid and mitigates the effects of drought in transgenic arabidopsis thaliana. J. Exp. Bot. 65, 1637–1649. doi: 10.1093/jxb/eru031 24609499 PMC3967093

[B149] Zafar S. A. Zaidi S. S. E. A. Gaba Y. Singla-Pareek S. L. Dhankher O. P. Li X.. (2020). Engineering abiotic stress tolerance via CRISPR/Cas-mediated genome editing. J. Exp. Bot. 71, 470–479. doi: 10.1093/jxb/erz476 31644801

[B150] Zhang Y. Xu J. Li R. Ge Y. Li Y. Li R. (2023). Plants’ Response to abiotic stress: mechanisms and strategies. Int. J. Mol. Sci. 24 (13), 10915. doi: 10.3390/ijms241310915 37446089 PMC10341657

[B151] Zhao C. Liu B. Piao S. Wang X. Lobell D. B. Huang Y.. (2017). Temperature increase reduces global yields of major crops in four independent estimates. Proc. Natl. Acad. Sci. U.S.A. 114, 9326–9331. doi: 10.1073/pnas.1701762114 28811375 PMC5584412

[B152] Zhao P. X. Miao Z. Q. Zhang J. Chen S. Y. Liu Q. Q. Xiang C.B. (2020). Arabidopsis MADS-box factor AGL16 negatively regulates drought resistance via stomatal density and stomatal movement. J. Exp. Bot. 71, 6092–6106. doi: 10.1093/jxb/eraa303 32594177

[B153] Zhao S. Zhang Q. Liu M. Zhou H. Ma C. Wang P. (2021). Regulation of plant responses to salt stress. Int. J. Mol. Sci. 22 (9), 4609. doi: 10.3390/ijms22094609 33924753 PMC8125386

